# Systematic review of Lonicerae Japonicae Flos: A significant food and traditional Chinese medicine

**DOI:** 10.3389/fphar.2022.1013992

**Published:** 2022-10-19

**Authors:** Senwang Zheng, Songtao Liu, Ajiao Hou, Song Wang, Yexin Na, Jianhua Hu, Hai Jiang, Liu Yang

**Affiliations:** Key Laboratory of Chinese Materia Medica, Heilongjiang University of Chinese Medicine, Ministry of Education, Harbin, China

**Keywords:** *Lonicerae Japonicae* Flos, botany, ethnopharmacology, phytochemistry, pharmacology, toxicology

## Abstract

Lonicerae Japonicae Flos has been used as a tea and medicine for more than 1,500 years. It has the functions of clearing heat, detoxification, and is often used to treat carbuncle, furuncle, throat arthralgia, erysipelas, heat-toxic blood dysentery, febrile fever. This paper summarizes the botany, ethnopharmacology, chemical composition and pharmacological action of Lonicerae Japonicae Flos from 1986 to 2022, and looks forward to the future research direction of Lonicerae Japonicae Flos. At present, the components isolated from Lonicerae Japonicae Flos include essential oils, organic acids, flavonoids, iridoids, saponins and other compounds. It has the effects of anti-inflammation, anti-virus, anti-bacteria, anti-oxidation, anti-tumor, protect liver and galltesticles, hypotensive, hypolipidemic, anti-thrombosis, anti-allergy, immune regulation and so on. It is often used in clinical treatment of diarrhea, hematochezia, febrile disease, exogenous wind-heat, and cold, swelling and toxin of carbuncle, sore throat and so on. The comprehensive evaluation of the quality of Lonicerae Japonicae Flos and the understanding of multi-target network pharmacology also need to be studied. As a kind of health food with high value, LJF is worthy of further promotion and development.

## Introduction

Lonicerae Japonicae Flos (LJF) is the dried bud or flower with initial blooming of *Lonicera japonica* Thunb.(Caprifoliaceae) (Hou and Jiang, 2013), it is used as food by Chinese people. LJF is also a commonly used traditional Chinese medicine with a long history, which is first listed in “Ming Yi Bie Lu” (名医别录) (A.D. 220-450) and listed as the top grade. LJF first appeared in Li Shizhen’s “Compendium of Materia Medica” and has been included in the Chinese Pharmacopoeia (Chinese Pharmacopoeia Commission, 2020). It tastes sweet and cold. Return to the meridians of the lung, heart and stomach. It has the functions of clearing away heat and detoxification, dispersing wind-heat and other functions, and is often used to treat carbuncle, furuncle, throat arthralgia, erysipelas, heat-toxic blood dysentery, wind-heat cold, febrile fever and other diseases. It is mainly distributed in Shandong, Henan and other places (Zhang et al., 2014).

Phytochemical studies show that the components isolated from LJF include essential oils, organic acids, flavonoids, iridoids, saponins and so on (Peng et al., 2000; Wang Y. et al., 2008; Guan et al., 2014; Wang L. et al., 2016; Ge et al., 2019). Pharmacological studies show that LJF has the effects of anti-oxidation, anti-tumor, anti-bacterial, anti-virus, anti-inflammation, anti-allergy, heat-clearing and detoxification, protecting liver and gallbladder, hypolipidemic, hypoglycemic, immune regulation and so on (Li, 2004; Li, 2006; Guo and Shi, 2009; Sun et al., 2010; Han et al., 2016; Wang et al., 2017; Bang et al., 2019; Fan et al., 2019; Wang et al., 2019).

LJF can be eaten raw or cooked with porridge or peach blossoms, which can boost the body’s immune system. More people take it as a tea drink, you can add mint, chrysanthemum, jasmine and so on while making tea, play the role of clearing heat and detoxifying. It is used to treat headache and sore throat. Frequent use of water extract of LJF gargle can also treat mouth sores and relieve sore throat.

In clinic, LJF is often used in combination with other drugs to form compound preparations, such as Xiao’ er Feire Kechuan Koufuye, Xiao’ er Yanbian Keli, Xiao’ er Tuire Heji, Xiao’ er Resuqing Koufuye, Xiao’ er Resuqing Tangjiang, Niuhuang Huadu Pian, Niuhuang Qinggan Jiaonang, Shuanghu Qinggan Keli and so on.

It has the functions of clearing heat and promoting diuresis, relieving sore throat, strengthening spleen and relieving diarrhea, removing toxin and relieving pain, dispelling wind pathogens, eliminating detumescence and resolving masses, resolving phlegm and promoting blood circulation. It is often used for diarrhea, abdominal pain, anorexia caused by damp-heat in children, fever caused by exogenous wind and heat, headache, sore throat, upper respiratory tract infection, runny nose, cough, dry stool, and the treatment of sores, mastitis and pain (Leung et al., 2008; Zhuang et al., 2013; Gao, 2018; Guang, 2018; Zhao, 2018).

LJF has high edible and medicinal value, before it was applied in thousand years. Its medicinal value and historical origin have been studied by doctors of past dynasties. This paper summarizes the botany, ethnopharmacology, chemical composition and pharmacological action of LJF, and summarizes all kinds of literature of LJF. It not only provides a reference for better development and utilization of LJF, but also provides guidance for the future research of LJF.

## Botany

**FIGURE 1:**
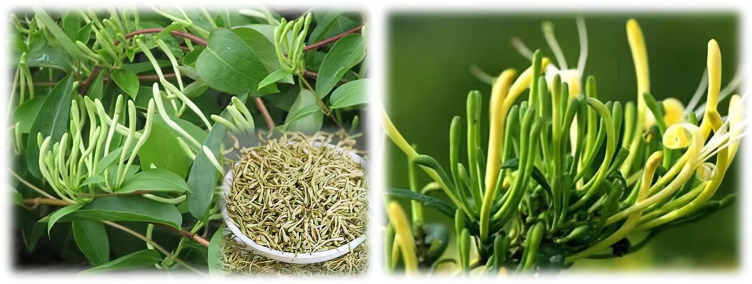
*Lonicera japonica* Thunb. and LJF.

According to the Chinese Pharmacopoeia (Edition 2020), Lonicerae Japonicae Flos (LJF) is the dried bud or flower with initial blooming of *Lonicera japonica* Thunb.. This plant enjoys a warm and humid climate, and enjoys sufficient sunshine, cold resistance, drought resistance and waterlogging resistance. It is suitable for growth at 20°C-30°C, with loose soil requirements and salt tolerance. It is suitable for cultivation in humus soil with deep and loose soil layers. It is mainly produced in Sichuan, Guangdong, Guangxi, Hunan, Guizhou, Yunnan and other places. The dried buds of *Lonicera japonica* Thunb. are long rod-shaped, thick at the top and thin at the bottom, slightly curved, 2-3 cm in length, 3 mm in diameter in the upper part and 1.5 mm in the lower part. The surface is yellowish-white or green-white (dark over time), covered with pubescent and glandular hairs. The base has a small green calyx, 5-lobed, triangular lobes, and glabrous. When the bud is cut open, there are 5 stamens and 1 pistil. The corolla is lip-shaped, and the androgens and pistils protrude like whiskers. The breath is fragrant, the taste is light and bitter. It is better for those whose flowers are not blooming, yellow and white and fat. From May to June, on a sunny morning, the buds are picked when the dew is dry, dried in the sun or in the shade on the stall, and pay attention to the rotation, otherwise it is easy to black. It should be protected from the hot sun. It should be kept in a highly dry and ventilated place to prevent insects and discoloration. (Wang et al., 2010; Kang et al., 2014). The pictures of *Lonicera japonica* Thunb. And Lonicerae Japonicae Flos are showed in Figure 1.

## Ethnopharmacology

LJF has been used in China for thousands of years and is recorded in many books. It has the function of clearing heat and detoxification. It can treat febrile disease, fever, blood dysentery, carbuncle, swelling, diarrhea, hemorrhoid leakage and so on. “Pin hui jing yao” (品汇精要) (A.D. 1505) records: “*Longicera Japonica* Thunb., is in blossom in March, with five flowers coming out, slightly fragrant, red peduncle, white flower at the beginning of blossom, and yellow after one or 2 days, so it is named as *Longicera Japonica* Thunb..” “Ben cao tong xuan” (本草通玄) (A.D. 1667) records: “LJF is effective for treating distension and dysentery, carbuncle and toxin, and wind deficiency. However, the world knows its efficacy in disinfection, not its efficacy in distending and draining wind and deficiency. It is always effective when used for this disease.” “Ben cao zheng” (本草正) (A.D. 1624) recorded: “LJF is good at poisoning, so it is an essential herb for treating carbuncle, gangrene, sore and tinea, waxberry and rheumatism. However, due to its slow action, it needs to be used more often, either by boiling with wine, or by pounding juice mixed with wine for quick drinking, or by grinding into pieces and mixing with wine for thick application.” “Ben jing feng yuan” (本经逢原) (A.D. 1695) records: “LJF is a holy drug for detoxicating, removing pus, tonifying in purging, and treating carbuncle, gangrene and collapse. However, it is not indicated for patients with qi deficiency and purulence clearing and loose stool due to lack of food.” “Dian nan ben cao” (滇南本草) (A.D. 1476) records: “LJF is cold in nature and tastes bitter. It can clear heat, remove various sores, carbuncle and carbuncle from the hair and back.” “Sheng cao yao xing bei yao” (生草药性备要) (A.D. 1711) records: “LJF can eliminate carbuncle, gangrene and furuncle, stop dysentery, wash malnutrition sore, skin blood heat.” “Ben cao bei yao” (本草备要) (A.D. 1694) records: “LJF can nourish blood to quench thirst to treat scabies.” “Chong qing tang sui bi” (重庆堂随笔) (A.D. 1808) records: “LJF can clear heat and promote diuresis, relievethe epidemic and the liver and gallbladder, and treat spasm, convulsion, epilepsy and other diseases.” “Chang yong zhong cao yao shou ce” (常用中草药手册) (1969) records: “LJF is capable of clearing heat and detoxicating, and treating exogenous fever, cough, enteritis, bacillary dysentery, measles, parotitis, septicemia, sore, furuncle and swelling toxin, appendicitis, traumatic infection, and infantile miliaria toxin. It can be made into herbal tea for prevent heatstroke, common cold and intestinal infectious diseases.”

### Common compatibility and application

In clinical application, LJF is often used in combination with other drugs. LJF compatible with Forsythiae Fructus, Arctii Fructus, Menthae Haplocalycis Herba, Schizonepetae Herba, used for surface antipyretic. LJF compatible with Scutellariae Radix, Coptidis Rhizoma, Paeoniae Radix Alba, Portulacae Herba, used for diarrhoea and stool bleeding. LJF compatible with Rehmanniaglutinosa(Gaetn.) Libosch. exFisch. et Mey., Scrophulariae Radix, Forsythiae Fructus, Lophatheri Herba, used to relieve heat. LJF combined with Scutellariae Radix, Angelicae Sinensis Radix, Paeoniae Radix Alba and Glycyrrhizae Radix Et Rhizoma is used for suppressing toxin, clearing heat and eliminating carbuncle. LJF combined with Violae Herba, Chrysanthemi Indici Flos, Taraxaci Herba, used for detoxification and treatment of sores.

The commonly used clinical compound preparations include Yinqiao San, Rendong San, Huichuang Jinyinhua San, Rendong Tang, Yinhua Tang, Shuanghuanglian Koufuye and Lianhua Qingwen Jiaonang (Table 1). Among them, Shuanghuanglian Koufuye and Lianhua Qingwen Jiaonang are the most widely used in clinic. They have strong effects on fever, cough, runny nose, headache, dry eye, sore throat, and muscle soreness caused by exogenous wind-fever and influenza. Other compound preparations also have the functions of clearing heat and detoxification, dispersing lung qi and dissipating phlegm, relieving pain, tonifying kidney and spleen, promoting diuresis and detumescence, calming nerves and relieving palpitation, nourishing blood and moistening skin, purging the lungs, relieving diarrhea and soothing the liver. The dosage forms involved include: water decoction, oral liquid preparation, syrup, tablet, capsule, granule, pill, spray, liniment, powder, paste, mixture and so on.

**TABLE 1:** The traditional clinical application and origin of LJF.

Preparation name	Dosage forms	Main compositions	Traditional and clinical uses	References
Yinqiao San	Powder	LJF, Forsythiae Fructus, Bitter Platycodon Grandiflorum, Menthae Haplocalycis Herba, Lophatheri Herba, Glycyrrhizae Radix Et Rhizoma, Schizonepetae Spica, Sojae Semen Praeparatum, Arctii Fructus	Cold fever	Wen Bing Tiao Bian (温病条辨)
Rendong San	Powder	LJF	Treat dysentery	Hui Zhi Tang Jing Yan Fang (惠直堂经验方)
Huichuang Jinyinhua San	Decoction	LJF, Astragali Radix, Glycyrrhizae Radix Et Rhizoma	Treat sores and ulcers	Huo Fa Ji Yao (活法机要)
Guihua Tang	Decoction	LJF, Angelicae Sinensis Radix	Treatment of carbuncle	Dong Tian Ao Zhi (洞天奥旨)
Rendong Tang	Decoction	LJF, Glycyrrhizae Radix Et Rhizoma	Treatment of carbuncle	Yi Xue Xin Wu (医学心悟)
Qingchang Tang	Decoction	LJF, Angelicae Sinensis Radix, Sanguisorbae Radix, Ophiopogonis Radix, Picriae Herba, Glycyrrhizae Radix Et Rhizoma, Coix Seed, Astragali Radix	Treatment of large intestine carbuncle	Dong Tian Ao Zhi (洞天奥旨)
Yinhua Tang	Decoction	LJF, Astragali Radix, Angelicae Sinensis Radix, Glycyrrhizae Radix Et Rhizoma, Trifoliate-Orange Immature Fruit Leaves	Treat sores and ulcers	Zhu Lin Nv Ke (竹林女科)
Rendong Tang	Decoction	LJF, Glycyrrhizae Radix Et Rhizoma, Glycinemax(L.)merr, Smilacis Glabrae Rhizoma	Reduce the toxicity	Wai Ke Shi Fa (外科十法)
Xiaoer Feire Kechuan Koufuye	Liquid preparation	Ephedrae Herba, Armeniacae Semen Amarum, Gypsum Fibrosum, Glycyrrhizae Radix Et Rhizoma, LJF, Forsythiae Fructus, Anemarrhenae Rhizoma, Scutellariae Radix, Isatidis Radix, Ophiopogonis Radix, Houttuyniae Herba	Clearing heat and detoxification, expelling lungs and resolving phlegm	Chinese Pharmacopoeia
Xiaoer Yanbian Keli	Granules	LJF, Belamcandae Rhizoma, Tinosporae Radix, Platycodonis Radix, Picriae Herba, Ophiopogonis Radix, Bovis Calculus Artifactus, Borneolum	Clearing heat and promoting pharynx, detoxifying and relieving pain	Chinese Pharmacopoeia
Xiaoer Tuire Heji	Mixture	Isatidis Folium, LJF, Gardeniae Fructus, Scutellariae Radix, Pheretima, Bupleuri Radix, Isatidis Radix, Forsythiae Fructus, Moutan Cortex, Lophatheri Herba, Paridis Rhizoma, Cynanchi Atrati Radix Et Rhizoma	Detoxification and pharynx	Chinese Pharmacopoeia
Xiaoer Tuire Koufuye	Liquid preparation			Chinese Pharmacopoeia
Xiaoer Tuire Keli	Granules			Chinese Pharmacopoeia
Xiaoer Resuqing Koufuye	Liquid preparation	Bupleuri Radix, Isatidis Radix, LJF, Forsythiae Fructus, Scutellariae Radix, Puerariae Lobatae Radix, Bubali Cornu, Rhei Radix Et Rhizoma	Clearing heat and detoxification, purging fire and promoting pharynx	Chinese Pharmacopoeia
Xiaoer Resuqing Keli	Granules			Chinese Pharmacopoeia
Xiaoer Resuqing Tangjiang	Syrup			Chinese Pharmacopoeia
Xiaoer Ganmaoning Tangjiang	Syrup	Menthae Haplocalycis Herba, Armeniacae Semen Amarum, Scutellariae Radix, Peucedani Radix, Gardeniae Fructus, Massa Medicata Fermentata, Phragmitis Rhizoma, Schizonepetae Spica, Arctii Fructus, Platycodonis Radix, Angelicae Dahuricae Radix, Crataegi Fructus, Hordei Fructus Germinatus, LJF, Forsythiae Fructus	Reduce fever and cough	Chinese Pharmacopoeia
Xiaoer Jiebiao Keli	Granules	LJF, Forsythiae Fructus, Arctii Fructus, Taraxaci Herba, Scutellariae Radix, Saposhnikoviae Radix, Perillae Folium, Schizonepetae Spica, Puerariae Lobatae Radix, Bovis Calculus Artifactus	Relieving the lung and relieving the surface, clearing heat and detoxification	Chinese Pharmacopoeia
Niuhuang Huadu Pian	Tablet	Arisaematis Rhizoma Preparatum, LJF, Glycyrrhizae Radix Et Rhizoma, Myrrha, Forsythiae Fructus, Angelicae Dahuricae Radix, Olibanum, Bovis Calculus Artifactus	Detoxification, detumescence and Relieve pain	Chinese Pharmacopoeia
Niuhuang Jingnao Pian	Tablet	Bovis Calculus Artifactus, Forsythiae Fructus, Coptidis Rhizoma, Taraxaci Herba, Cinnabaris, Calcined Magnet, Pige 'S Bile, Realgar, Trichosanthis Radix, Rehmanniae Radix, Scrophulariae Radix, Rhei Radix Et Rhizoma, Glycyrrhizae Radix Et Rhizoma, LJF, Scutellariae Radix, Gypsum Ustum, Margarita, Haliotidis Concha, Haematitum, Borneolum Syntheticum, Ophiopogonis Radix, Puerariae Lobatae Radix, Isatidis Radix, Gardeniae Fructus, Curcumae Radix	Clearing heat and detoxification	Chinese Pharmacopoeia
Niuhuang Qinggong Wan	Pill	Bovis Calculus Artifactus, Scutellariae Radix, Trichosanthis Radix, Rhei Radix Et Rhizoma, Rehmanniae Radix, Curcumae Radix, Realgar, Cinnabaris, LJF, Ophiopogonis Radix, Nelumbinis Plumula, Glycyrrhizae Radix Et Rhizoma, Gardeniae Fructus, Forsythiae Fructus, Scrophulariae Radix, Powerdered Buffalo Horn	Clearing heat and detoxification	Chinese Pharmacopoeia
		Extract, Borneolum Syntheticum, Artificial Musk		
Niuhuang Qinggan Jiaonang	Capsule	Scutellariae Radix, LJF, Forsythiae Fructus, Bovis Calculus Artifactus, Margaritifera Concha	Clearing heat and detoxification	Chinese Pharmacopoeia
Shuanghu Qinggan Keli	Granules	LJF, Polygoni Cuspidati Rhizoma	Clearing heat, resolving phlegm and activating blood circulation	Chinese Pharmacopoeia
		Et Radix, Coptidis Rhizoma, Trichosanthis Fructus, Hedyotis Diffusa, Taraxaci Herba, Salviae Miltiorrhizae		
		Radix Et Rhizoma, Chrysanthemi Indici Flos, Violae Herba, Pinelliae Rhizoma Praeparatum, Aurantii Fructus Immaturus, Glycyrrhizae Radix Et Rhizoma		
Shuanghuanglian Koufuye	Liquid preparation	LJF, Scutellariae Radix, Forsythiae Fructus	Clearing heat and detoxification	Chinese Pharmacopoeia
Shuanghuanglian Pian	Tablet			Chinese Pharmacopoeia
Shuanghuanglian Shuan	Suppository			Chinese Pharmacopoeia
Shuanghuanglian Jiaonang	Capsule			Chinese Pharmacopoeia
Shuanghuanglian Keli	Granules			Chinese Pharmacopoeia
Shuanghuanglian Diyanji	Eye drops			Chinese Pharmacopoeia
Qidong Yixin Koufuye	Liquid preparation	Scutellariae Radix, Ophiopogonis Radix, Ginseng Radix Et Rhizoma, Poria, Rehmanniae Radix, Testudinis Carapax Et Plastrum, Fluorite, Cinnamomi Ramulus, Epimedii Folium, LJF, Salviae Miltiorrhizae Radix Et Rhizoma, Curcumae Radix, Aurantii Fructus	Soothe the nerves	Chinese Pharmacopoeia
Qidong Yixin Keli	Granules			Chinese Pharmacopoeia
Kegan Liyuan Koufuye	Liquid preparation	LJF, Scutellariae Radix, Schizonepetae Spica, Gardeniae Fructus, Forsythiae Fructus, Scrophulariae Radix, Bombyx Batryticatus, Rehmanniae Radix, Belamcandae Rhizoma, Platycodonis Radix, Menthae Haplocalycis Herba, Cicadae Periostracum, Saposhnikoviae Radix, Glycyrrhizae Radix Et Rhizoma	Detoxification	Chinese Pharmacopoeia
Lianhua Qingwen Pian	Tablet	Forsythiae Fructus, LJF, Ephedrae Herba, Armeniacae Semen Amarum, Gypsum Fibrosum, Isatidis Radix, Dryopteridis Crassirhizomatis Rhizoma, Houttuyniae Herba, Pogostemonis Herba, Rhei Radix Et Rhizoma, Rhodiolae Crenulatae Radix Et Rhizoma, L-Menthol, Glycyrrhizae Radix Et Rhizoma	Detoxification and Protect the lungs	Chinese Pharmacopoeia
Lianhua Qingwen Jiaonang	Capsule			Chinese Pharmacopoeia
Lianhua Qingwen Keli	Granules			Chinese Pharmacopoeia
Kanggu Suiyan Pian	Tablet	LJF, Taraxaci Herba, Violae Herba, Scutellariae Barbatae Herba, Pulsatilla! Radix, Oldenlandia Diffusa	Clearing heat, etumescence and detoxification	Chinese Pharmacopoeia
Kanggan Koufuye	Liquid preparation	LJF, Paeoniae Radix Rubra, Dryopteridis Crassirhizomatis	Clearing heat and detoxification	Chinese Pharmacopoeia
Kanggan Keli	Granules	Rhizoma		Chinese Pharmacopoeia
Liyan Jiedu Keli	Granules	Isatidis Radix, LJF, Forsythiae Fructus, Menthae Haplocalycis Herba, Arctii Fructus, Crataegi Fructus, Platycodonis Radix, Bombyx Batryticatus, Scrophulariae Radix, Scutellariae Radix, Rehmanniae Radix, Trichosanthis Radix, Rhei Radix Et Rhizoma, Fritillariae Thunbergii Bulbus, Ophiopogonis Radix	Protect lungs and throat	Chinese Pharmacopoeia
Lidan Pian	Tablet	Rhei Radix Et Rhizoma, LJF, Lysimachiae Herba, Aucklandiae Radix, Anemarrhenae Rhizoma, Isatidis Folium, Bupleuri Radix, Paeoniae Radix Alba, Scutellariae Radix, Natrii Sulfas, Artemisiae Scopariae Herba	Protect the liver and relieve pain	Chinese Pharmacopoeia
Qingguo Wan	Pill	Canarii Fructus, LJF, Scutellariae Radix, Menispermi Rhizoma, Ophiopogonis Radix, Scrophulariae Radix, Paeoniae Radix Alba, Platycodonis Radix	Reduce swelling and relieve pain	Chinese Pharmacopoeia
Badu Gao	Ointment	LJF, Forsythiae Fructus, Rhei Radix Et Rhizoma, Platycodonis Radix, Rehmanniae Radix, Gardeniae Fructus, Phellodendri Chinensis Cortex, Scutellariae Radix, Paeoniae Radix Rubra, Angelicae Sinensis Radix, Chuanxiong Rhizoma, Angelicae Dahuricae Radix, Ampelopsis Radix, Momordicae Semen, Ricini Semen, Scrophulariae Radix, Atractylodis Rhizoma, Scolopendra, Camphor, Manis Squama, Myrrha, Catechu, Olibanum, Hydrargyri Oxydum Rubrum, Draconis Sanguis, Calomelas	Clearing heat and detoxification, Promoting blood circulation and detumescence	Chinese Pharmacopoeia
Shenyanshu Pian	Tablet	Atractylodis Rhizoma, Poria, Lalang Grass Rhizome, Stephaniae Tetrandrae Radix, Ginseng Radix Et Rhizoma, Polygonati Rhizoma, Cuscutae Semen, Lycii Fructus, LJF, Taraxaci Herba	Tonifying the kidney and invigorating the spleen, Diuresis and detumescence	Chinese Pharmacopoeia
Jinbei Tankeqing Keli	Granules	Fritillariae Thunbergii Bulbus, LJF, Peucedani Radix, Armeniacae Semen Amarum, Mori Cortex, Platycodonis Radix, Belamcandae Rhizoma, Ephedrae Herba, Chuanxiong Rhizoma, Glycyrrhizae Radix Et Rhizoma	Tonifying lung and kidney	Chinese Pharmacopoeia
Jinqi Jiangtang Pian	Tablet	Coptidis Rhizoma, Astragali Radix, LJF	Clear heat	Chinese Pharmacopoeia
Jinyinhua Lu	Spray	LJF	Clearing heat and detoxification	Chinese Pharmacopoeia
Jinpu Jiaonang	Capsule	Bovis Calculus Artifactus, LJF, Scolopendra, Manis Squama, Bufonis Venenum, Taraxaci Herba, Scutellariae Barbatae Herba, Cremastrae Pseudobulbus Pleiones Pseudobulbus, Curcumae Rhizoma, Hedyotis Diffusa, Sophorae Flavescentis Radix, Solanum Nigrum, Margarita, Rhei Radix Et Rhizoma, Airpotato Mot Rhizome, Olibanum, Myrrha, Corydalis Rhizoma, Carthami Flos, Pinelliae Rhizoma Praeparatum Cum Zingibere Et Alumine, Codonopsis Radix, Astragali Radix, Acanthopanacis Senticosi Radix Et Rhizoma Seu Caulis, Amomi Fructus	Clearing heat and detoxification, reducing swelling and relieving pain, Resolving phlegm	Chinese Pharmacopoeia
Jinsang Kaiyin Wan	Pill	LJF, Forsythiae Fructus, Scrophulariae Radix, Isatidis Radix, Paeoniae Radix Rubra, Scutellariae Radix, Mori Folltfm, Chrysanthemi Flos, Peucedani Radix, Armeniacae Semen Amarum, Arctii Fructus, Alismatis Rhizoma, Sterculiae Lychnophorae Semen, Bombyx Batryticatus, Cicadae Periostracum, Oroxyli Semen	Clearing heat and detoxification, and promoting pharynx.	Chinese Pharmacopoeia
Zhizi Jinhua Wan	Pill	Gardeniae Fructus, Coptidis Rhizoma, Scutellariae Radix, Phellodendri Chinensis Cortex, Rhei Radix Et Rhizoma, LJF, Anemarrhenae Rhizoma, Trichosanthis Radix	Clearing heat and detoxification	Chinese Pharmacopoeia
Fufang Daqingye He Ji	Mixture	Isatidis Folium, LJF, Notopterygii Rhizoma Et Radix, Bistortae Rhizoma, Rhei Radix Et Rhizoma	Clearing heat, detoxification, cholagogic and detumescence	Chinese Pharmacopoeia
Fufang Qinlan Koufuye	Liquid preparation	LJF, Scutellariae Radix, Forsythiae Fructus, Isatidis Radix	Clearing heat and detoxification	Chinese Pharmacopoeia
Fufang Kushen Changyankang Pian	Tablet	Sophorae Flavescentis Radix, Coptidis Rhizoma, Scutellariae Radix, Paeoniae Radix Alba, Plantaginis Semen, LJF, Glycyrrhizae Radix Et Rhizoma, Belladonna Liquid Extract	Relieving diarrhea	Chinese Pharmacopoeia
Fufang Jinhuanglian Keli	Granules	Forsythiae Fructus, Taraxaci Herba, Scutellariae Radix, LJF, Isatidis Radix	Clear heat and dipharynx	Chinese Pharmacopoeia
Fufang Yuxingcao Pian	Tablet	Houttuyniae Herba, Scutellariae Radix, Isatidis Radix, Forsythiae Fructus, LJF	Clearing heat and detoxification	Chinese Pharmacopoeia
Fufang Huangbaiye Tuji	Paint	Forsythiae Fructus, Phellodendri Chinensis Cortex, LJF, Taraxaci Herba, Scolopendra	Clearing heat and detoxification, detumescence and detoxification	Chinese Pharmacopoeia
Fufang Huangbai Ye	Liquid preparation			Chinese Pharmacopoeia
Chaiyin Koufuye	Liquid preparation	Bupleuri Radix, LJF, Scutellariae Radix, Puerariae Lobatae Radix, Schizonepetae Herba, Platycodonis Radix, Armeniacae Semen Amarum, Menthae Haplocalycis Herba, Houttuyniae Herba	Heat and detoxification, promoting pharynx and relieving cough	Chinese Pharmacopoeia
Langchuang Wan	Pill	LJF, Taraxaci Herba, Rehmanniae Radix, Glycyrrhizae Radix Et Rhizoma, Paeoniae Radix Rubra, Salviae Miltiorrhizae Radix Et Rhizoma, Persicae Semen, Cicadae Periostracum, Forsythiae Fructus, Coptidis Rhizoma, Rhei Radix Et Rhizoma, Scolopendra, Angelicae Sinensis Radix, Scrophulariae Radix, Carthami Flos, Fritillariae Thunbergii Bulbus	Detoxification and activating blood	Chinese Pharmacopoeia
Xiaoyin Pian	Tablet	Rehmanniae Radix, Paeoniae Radix Rubra, Sophorae Flavescentis Radix, Cicadae Periostracum, Saposhnikoviae Radix, Carthami Flos, Moutan Cortex	Clearing heat and cooling blood, nourishing blood and moisturizing skin	Chinese Pharmacopoeia
Xiaoyin Jiaonang	Capsule	Angelicae Sinensis Radix, LJF, Arctii Fructus, Dictamni Cortex, Isatidis Folium		Chinese Pharmacopoeia
Yinqiao Shuangjie Shuan	Suppository	Forsythiae Fructus, LJF, Scutellariae Radix, Folium Syringae,	Treat the lungs	Chinese Pharmacopoeia
Yinqiao San	Powder	LJF, Forsythiae Fructus, Platycodonis Radix, Menthae Haplocalycis Herba, Sojae Semen Praeparatum, Lophatheri Herba, Arctii Fructus, Schizonepetae Herba, Phragmitis Rhizoma, Glycyrrhizae Radix Et Rhizoma	Clearing heat and detoxification	Chinese Pharmacopoeia
Yinqiao Jiedu Wan	Pill			Chinese Pharmacopoeia
Yinqiao Jiedu Pian	Tablet			Chinese Pharmacopoeia
Yinqiao Jiedu Jiaonang	Capsule			Chinese Pharmacopoeia
Yinqiao Jiedu Keli	Granules			Chinese Pharmacopoeia
Biyuan Wan	Pill	Xanthii Fructus, Magnoliae Flos, LJF, Rubiae Radix Et Rhizoma, Chrysanthemi Indici Flos	Releasing lungs, clearing heat,detoxification and relieving pain	Chinese Pharmacopoeia
Biyuan Pian	Pill			Chinese Pharmacopoeia
Longqing Pian	Tablet	Alismatis Rhizoma, Plantaginis Semen, Dahurian Patrinia Herb, LJF, Moutan Cortex, Hedyotis Diffusa, Paeoniae Radix Rubra, Agrimoniae Herba, Coptidis Rhizoma, Phellodendri Chinensis Cortex	Clearing heat and detoxification	Chinese Pharmacopoeia

In addition, LJF is also used in the fields of animal husbandry, food, health products, daily necessities and cosmetics and applies for several patents. Such as moxa pig feed and preparation method (CN201911004525.1), preparation method and application of Chinese herbal medicine preparation for treating live pig viral enteritis (CN201910867827.5), nutritious hot pot seasoning and preparation method (CN201910862216.1), LJF flavored apple canned (CN201810679783.9), a herbal tea whose main ingredients are Hordeum vulgare Linn. var. nudum Hook.f., Eleocharis dulcis (Burm. f.) Trin. and LJF. (CN201911234063.2), a preparation method of LJF ginseng health herbal tea (CN201911172448.0), a preparation method of bagged asparagus LJF substitute meal solution (CN201911203081.4), a Chinese herbal medicine children’s toothpaste (CN201810649037.5), a Humei LJF facial mask (CN201930421728.5), an anti-aging and anti-wrinkle essence solution (CN201910895717.X), etc.

To date, LJF has been used in a variety of ethnic pharmaceutical formulations and foods. There are more than one hundred prescriptions involving LJF, among which there are a large number of prescriptions with LJF as the monarch drug. Among them, 62 proprietary Chinese patent medicines are sold in the medicine market. With this long history and these varied applications, LJF actually has a good curative effect on diseases. However, undiscovered active ingredient and molecular mechanism, unified quality of LJF, which all prevent LJF from being widely used in modern clinic. We summarize the identified components and pharmacological studies of LJF in the following sections, hope to provide a material basis for further development and utilization of LJF.

## Phytochemistry

**FIGURE 2:**
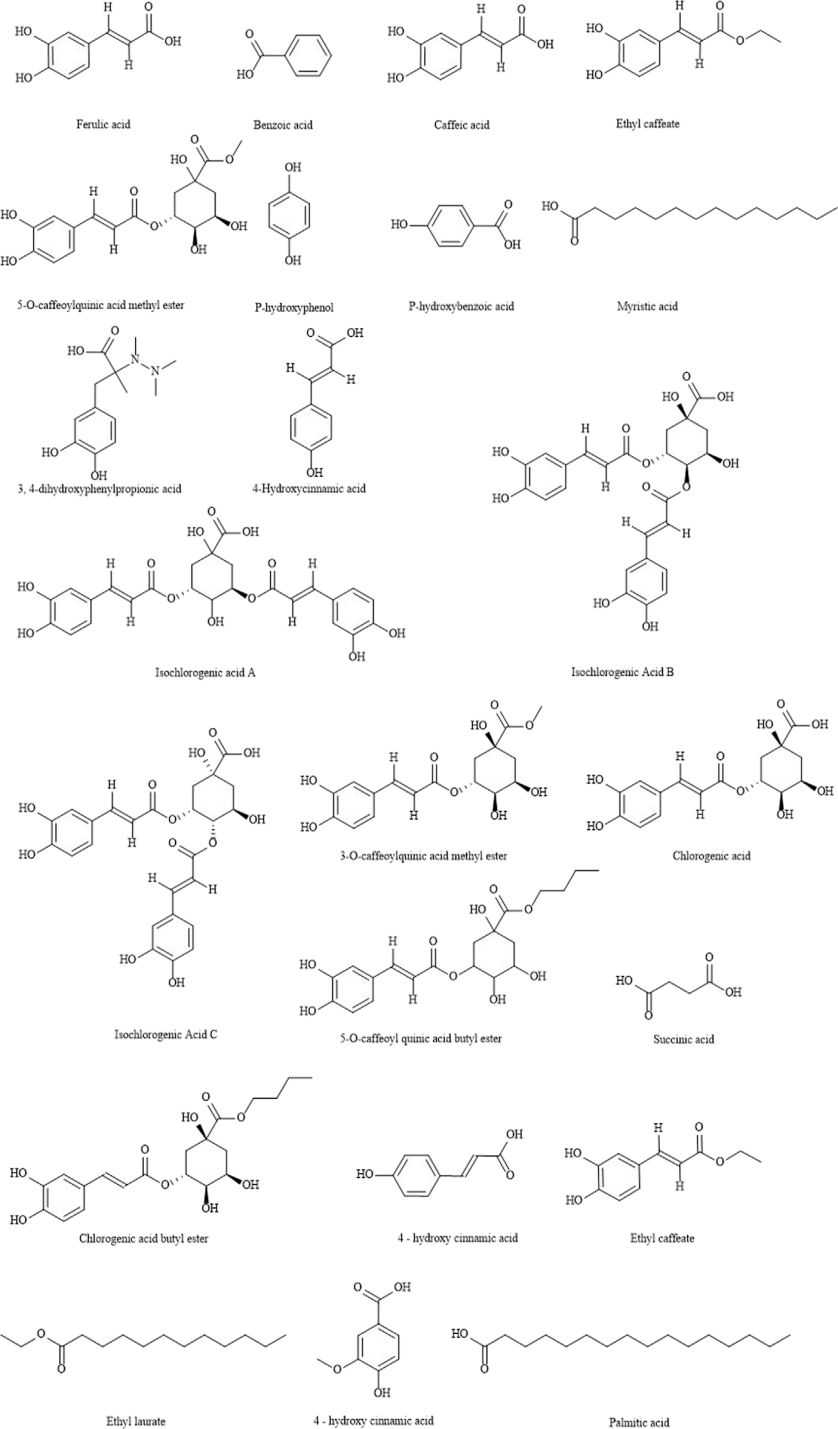
The structures of organic acids from LJF.

**FIGURE 3:**
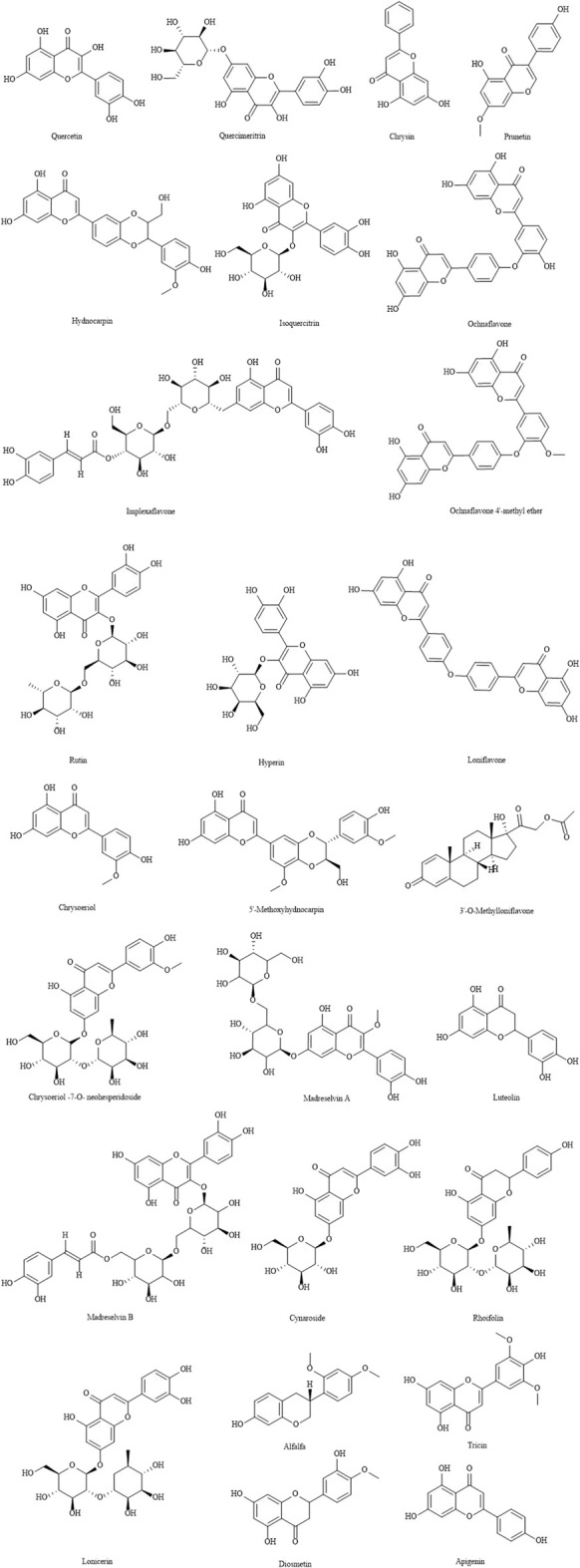
The structures of flavonoids from LJF.

**FIGURE 4:**
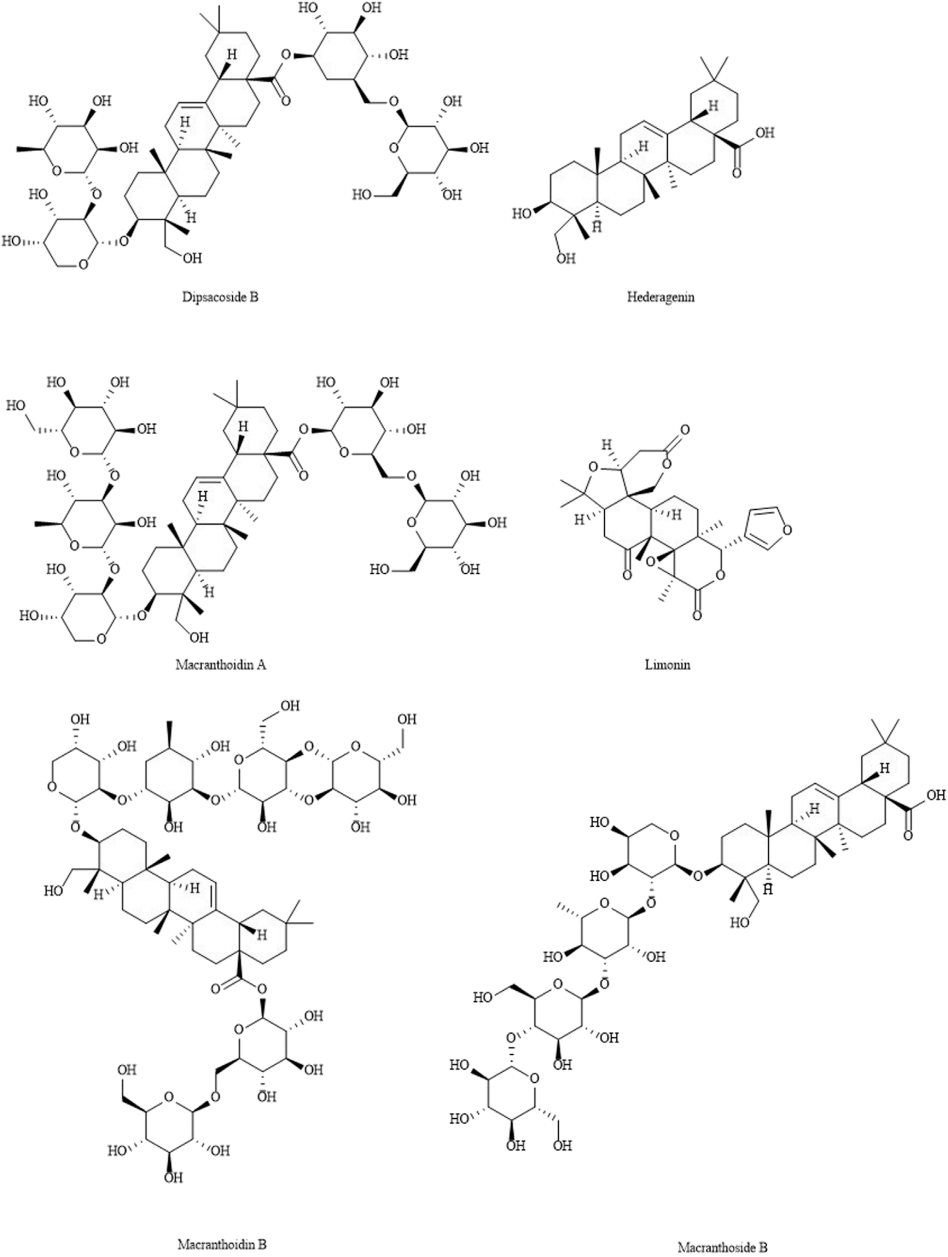
The structures of triterpenes and triterpenoid saponins from LJF.

**FIGURE 5:**
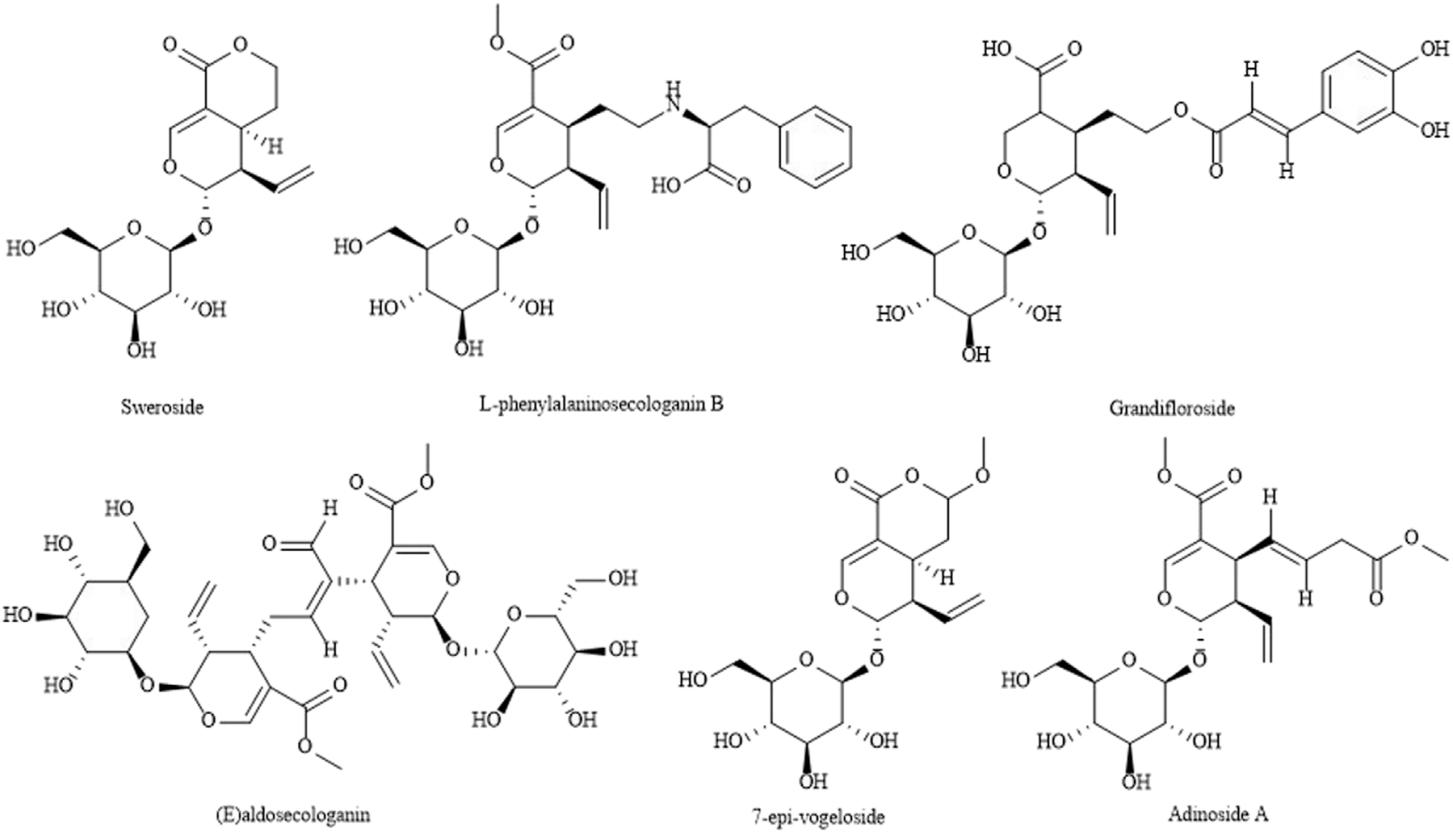
The structures of iridoids LJF.

**FIGURE 6:**
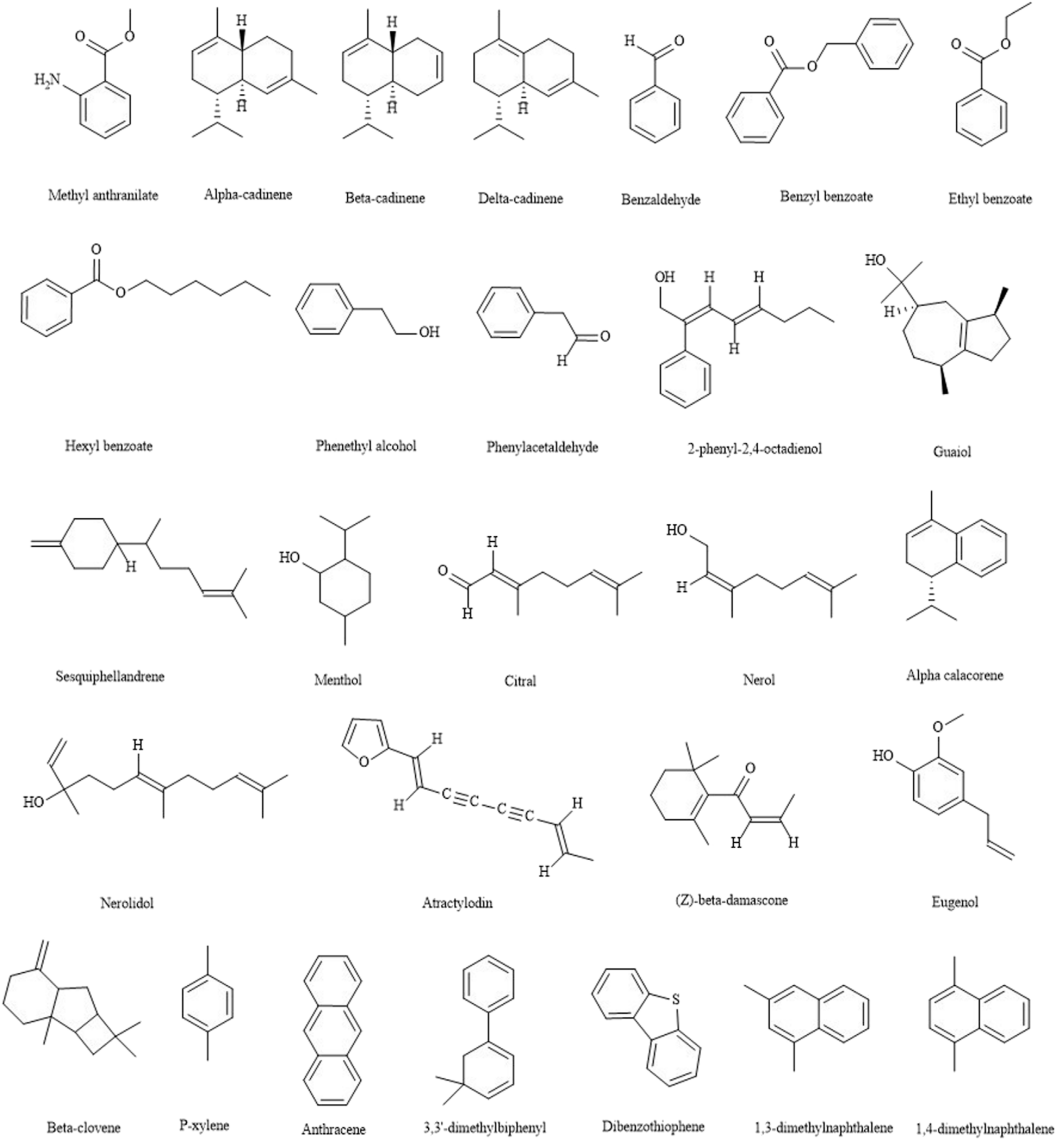
The main structures of essential oils from LJF.

LJF contains essential oils, organic acids, flavonoids, iridoids, saponins, trace elements and so on. In this paper, the chemical constituents of LJF are summarized. The structure of the main chemical components of LJF is shown in Figures 2–6.

### Organic acids

Organic acids are the main components of LJF. The 2020 edition of Chinese Pharmacopoeia takes chlorogenic acid as the index component to control the quality of LJF, and stipulates that the content of chlorogenic acid in LJF should not be less than 1.5%. So far, about 44 kinds of organic acids have been isolated from LJF (Table 2) (Iwahashi et al., 1986; Choi et al., 2007; Qi et al., 2009; Song et al., 2014; Chen et al., 2015; Yang et al., 2016; Liu et al., 2018; Tan et al., 2018; Wu et al., 2019; Yang, 2019), which can be used as antioxidation, anticancer, liver protection, immune regulation, bacteriostasis, anti-virus, relieving cough and asthma and so on. The structures of organic acids are shown in Figure 2.

**TABLE 2:** Organic acid components in LJF.

NO.	Components	References
1	Ferulic acid	Chen et al. (2015)
2	4-ferulic acid	Liu et al. (2018)
3	5-ferulic acid	Liu et al. (2018)
4	Benzoic acid	Wu et al. (2019)
5	1,2,4-pyrogallol	Yang (2019)
6	Caffeic acid	Choi et al. (2007)
7	Ethyl caffeate	Wu et al. (2019)
8	5-O-caffeoylquinic acid methyl ester	Wu et al. (2019)
9	P-hydroxyphenol	Chen et al. (2015)
10	P-hydroxybenzoic acid	Wu et al. (2019)
11	Myristic acid	Song et al. (2014)
12	1, 3-dihydroquinic acid	Liu et al. (2018)
13	1, 5-dihydroquinic acid	Chen et al. (2015)
14	3, 4-dihydroxyphenylpropionic acid	Tan et al. (2018)
15	Isochlorogenic acid A	Iwahashi et al. (1986)
16	Isochlorogenic Acid B	Iwahashi et al. (1986)
17	Isochlorogenic Acid C	Iwahashi et al. (1986)
18	3, 5-dio-O-caffeinoquinic acid methyl ester	Chen et al. (2015)
19	3, 4-dio-O-caffeinoquinic acid methyl ester	Chen et al. (2015)
20	4, 5-dio-O-caffeinoquinic acid methyl ester	Chen et al. (2015)
21	3, 5-dio-O-caffeoquinillate ethyl ester	Chen et al. (2015)
22	3, 4-dio-O-caffeoquinillate ethyl ester	Chen et al. (2015)
23	3, 5-dio-O-bari-quinillate	Chen et al. (2015)
24	4-Hydroxycinnamic acid	Chen et al. (2015)
25	5-O-caffeoquinic acid	Qi et al. (2009)
26	4-O-caffeoquinic acid	Qi et al. (2009)
27	1-O-caffeoquinic acid	Tan et al. (2018)
28	3-O-caffeoylquinic acid methyl ester	Chen et al. (2015)
29	5-O-caffeoyl quinic acid butyl ester	Chen et al. (2015)
30	Succinic acid	Chen et al. (2015)
31	Chlorogenic acid	Yang et al. (2016)
32	Chlorogenic acid butyl ester	Chen et al. (2015)
33	Bis -(2- methyl propyl) phthalate	Tan et al. (2018)
34	Lonfuranacid A	Wu et al. (2019)
35	Lonfuranacid B	Wu et al. (2019)
36	4 - hydroxy cinnamic acid	Chen et al. (2015)
37	4 - hydroxy cinnamic acid methyl ester	Chen et al. (2015)
38	Cinnamic acid	Chen et al. (2015)
39	Ethyl laurate	Tan et al. (2018)
40	Tetrapedicacid B	Wu et al. (2019)
41	Vanillic acid	Wu et al. (2019)
42	4-O- vanilic acid -D-6-O- benzoylpyranosyl group	Song et al. (2014)
43	2(E) -3-ethoxy acrylic acid	Liu et al. (2018)
44	Palmitic acid	Liu et al. (2018)

### Flavonoids

Flavonoids are one of the main active components of LJF. According to the 2020 edition of Chinese Pharmacopoeia, the flavonoids in LJF should not be less than 0.050%. At present, there are about 62 kinds of flavonoids found in LJF (Table 3) (Son et al., 1992; Lee et al., 1995; Ren et al., 2008; Song et al., 2014; Chen et al., 2015; Liu et al., 2018; Tan et al., 2018; Wu et al., 2019; Yang, 2019), which have antibacterial, antiviral, anti-tumor, anti-oxidation, anti-inflammatory and analgesic activities, protecting liver and gallbladder, hypolipidemic, immune regulation, antitussive and expectorant, anti-allergy and so on. The structures of Flavonoids are shown in Figure 3.

**TABLE 3:** Flavonoids in LJF.

NO.	Components	References
1	Chrysin	Chen et al. (2015)
2	5,3 ′-Dimethoxyluteolin	Liu et al. (2018)
3	Prunetin	Chen et al. (2015)
4	Flavoyadorininb	Song et al. (2014)
5	Hydnocarpin	Son et al. (1992)
6	Yellow Querletoside B	Chen et al. (2015)
7	Quercetin	Yang (2019)
8	Isoquercitrin	Liu et al. (2018)
9	Quercetin - 3-O-α-L-Pyran Rhamnoside	Wu et al. (2019)
10	Quercimeritrin	Liu et al. (2018)
11	Implexaflavone	Song et al. (2014)
12	Ochnaflavone	Chen et al. (2015)
13	Ochnaflavone 4′-methyl ether	Chen et al. (2015)
14	Rutin	Ren et al. (2008)
15	Loniflavone	Chen et al. (2015)
16	Hyperin	Tan et al. (2018)
17	Chrysoeriol	Chen et al. (2015)
18	Chrysoeriol -7-O- neohesperidoside	Liu et al. (2018)
19	Chrysoeriol 7-O-β-D-glucuronopyranosyl	Chen et al. (2015)
20	3' -Methoxyluteolin	Liu et al. (2018)
21	5′-Methoxyhydnocarpin	Wu et al. (2019)
22	3′-O-Methylloniflavone	Chen et al. (2015)
23	Madreselvin A	Song et al. (2014)
24	Madreselvin B	Song et al. (2014)
25	Luteolin	Yang (2019)
26	Cynaroside	Liu et al. (2018)
27	Luteolin - 7- O-α-D-Glucoside	Liu et al. (2018)
28	Luteolin -7-O-β-D - Glucoside	Tan et al. (2018)
29	Luteolin -7-O-β-D - Galactoside	Liu et al. (2018)
30	Luteolin -5-O-β-D -Glucoside	Liu et al. (2018)
31	Luteolin -7-O- Neohesperidoside	Chen et al. (2015)
32	5-Hydroxy-6, 7, 8, 4' -Tetramethoxy Flavone	Wu et al. (2019)
33	5-Hydroxy-7, 4-Dimethoxyflavone Quercetin	Wu et al. (2019)
34	5-Hydroxy-7, 3′, 4' -Trimethoxy Flavones	Tan et al. (2018)
35	5-Hydroxy-7,3′,4′,5′-Tetramethoxy Flavone	Chen et al. (2015)
36	Apigenin-7-O-α-L-Rhamnoside	Wu et al. (2019)
37	Apigenin-7-O-β-D-Glucoside	Chen et al. (2015)
38	Apigenin-5-O-β-D-Glucoside	Chen et al. (2015)
39	Rhoifolin	Song et al. (2014)
40	Lonicerin	Lee et al. (1995)
41	Kaempferol-3-O-Rutinoside	Tan et al. (2018)
42	Kaempferol-3-O-β-D-Glucopyranoside	Wu et al. (2019)
43	Kaempferol-3-O-β-D-Glucoside	Chen et al. (2015)
44	Kaempferol-7-O-β-D-Glucoside	Chen et al. (2015)
45	5,7,3′,4′-Tetrahydroxyflavonol-3-O-β-D-Glucoside	Chen et al. (2015)
46	5,7,4′-Trihydroxy-8-Methoxyflavone	Chen et al. (2015)
47	3 ′, 4′, 5 ′, 5,7-Pentamethoxyflavone	Tan et al. (2018)
48	Alfalfa-7-O- Neohesperidoside	Liu et al. (2018)
49	Alfalfa	Liu et al. (2018)
50	Tricin	Chen et al. (2015)
51	Alfalfa-7-O-β-D-Glucoside	Chen et al. (2015)
52	Geranin-7- O-β-D-Glucoside	Chen et al. (2015)
53	Diosmetin	Chen et al. (2015)
54	Diosmin	Chen et al. (2015)
55	Isorhamnetin	Wu et al. (2019)
56	Isorhamnetin-3-O-Rutinoside	Chen et al. (2015)
57	Isorhamnetin-7-O-β-D-Glucoside	Chen et al. (2015)
58	Isorhamnetin-3-O-β-D-Glucoside	Chen et al. (2015)
59	Apigenin	Tan et al. (2018)
60	Ethylshikonin Dimethyl Acetal	Wu et al. (2019)
61	Astragalin	Tan et al. (2018)
62	Apigenin-7-O-L-Rhamnoside	Chen et al. (2015)

### Triterpenes and triterpenoid saponins

Triterpenes and triterpenoid saponins are more active components in LJF. So far, more than 35 species of triterpenoids have been isolated from LJF (Table 4) (Chen et al., 2015; Liu et al., 2018; Wu et al., 2019; Yang, 2019). It has anti-inflammatory, anti-tumorous, antibacterial, antiviral and anti-fertility activities. The structures of triterpenes and triterpenoid saponins are shown in Figure 4.

**TABLE 4:** Triterpenes and triterpene saponins in LJF.

NO.	Components	References
1	3-O-β-D-glucopyranosyl (1 → 4) -β-D-glucopyranosyl (1 → 3) -α-L-rhamnopyranosyl (1 → 2) -α- L-arabinose-Changchun saponin-28-O-β-D-glucopyranosyl (1 → 6) -β-D-glucopyranosyl ester	Liu et al. (2018)
2	3-O-α-L-Rhamnopyranosyl (1 → 2) -α-L-arabinopyranosyl-vinchunsaponin-28-O-β-D-xylopyranose (1 → 6 ) -β-D-glucopyranose ester	Liu et al. (2018)
3	3-O-α-L-Rhamnopyranosyl (1 → 2) -α-L-arabinopyranosyl-Changchunsaponin-28-O-β-D-glucopyranosyl (1 → 6 ) -β-D-glucopyranose ester	Liu et al. (2018)
4	3-O-α-L-Rhamnopyranosyl (1 → 2) -α-L-arabinopyranosyl-Changchunsaponin-28-O-α-L- (1 → 2) -β- D-xylopyranose (1 → 6) -β-D-glucopyranosyl ester	Chen et al. (2015)
5	3-O-α-L-Rhamnopyranosyl (1 → 2) -α-L-arabinopyranosyl-Changchunsaponin-28-O-α-L-Rhamnopyranosyl → 2) -β-D-xylopyranose (1 → 6) -β-D-glucopyranosyl ester	Liu et al. (2018)
6	3-O-β-D-glucopyranosyl (1 → 3) -α-L rhamnopyranosyl (1 → 2) -α-L-arabinopyranosyl-Changchunsaponin-28-O -β-D-glucopyranosyl (1 → 6) -β-D-glucopyranosyl ester	Liu et al. (2018)
7	3-O-α-L-arabinopyranosyl-Changchun saponin-28-O-α-L-rhamnopyranosyl (1 → 2) -β-D-xylopyranose 6) -β-D-glucopyranose ester	Chen et al. (2015)
8	3-O-α-L-Rhamnosyl (1 → 2) -α-L-arabinopyranosyl-vinchunsaponin-28-O-6-acetoxy-β-D-glucopyranosyl- (1 → 6) -β-D-glucopyranoside	Chen et al. (2015)
9	3-O-α-L-arabinose-oleanolic acid-28-O-β-D-glucopyranosyl (1 → 6) -β-D-glucopyranosyl ester	Chen et al. (2015)
10	3-O-α-L-Rhamnopyranosyl (1 → 2) -α-L-arabinopyranosyl-oleanolic acid	Chen et al. (2015)
11	3-O-β-D-glucopyranosyl-Changchun saponin-28-O-β-D-glucopyranosyl (1 → 2) β-D-xylopyranosyl (1 → 6) -β -D-glucopyranoside	Chen et al. (2015)
12	3-O-α-L-arabinopyranosyl-oleanolic acid-28-O-β-D-glucopyranosyl (1 → 6) -β-D-glucopyranoside	Chen et al. (2015)
13	3-O-α-L-Rhamnosylglycosyl (1 → 2) -α-L-arabinyl arabinosyl-oleanolic acid-28-O-β-D-glucopyranosyl (1 → 6) -β-D-glucopyranoside	Chen et al. (2015)
14	Dipsacoside B	Wu et al. (2019)
15	Changchun saponin-3-O-α-L-rhamnopyranosyl (1 → 2) -α-L-arabinopyranoside	Liu et al. (2018)
16	Hederagenin	Wu et al. (2019)
17	Macranthoidin A	Wu et al. (2019)
18	Macranthoidin B	Wu et al. (2019)
19	Kalopanax saponin H	Wu et al. (2019)
20	Macranthoside B	Wu et al. (2019)
21	Japonicaside C	Wu et al. (2019)
22	Leontoside A	Chen et al. (2015)
23	Limonin	Chen et al. (2015)
24	Akebia saponin D	Wu et al. (2019)
25	Xyloside F	Chen et al. (2015)
26	Oleanic acid	Yang (2019)
27	Oleanolic acid-28-O-α-L-rhamnopyranosyl (1 → 2) -β-D-xylopyranosyl (1 → 6) -β-D-glucopyranoside	Chen et al. (2015)
28	Lonicin A	Chen et al. (2015)
29	Lonicin B	Chen et al. (2015)
30	Lonicin C	Chen et al. (2015)
31	Lonicin D	Chen et al. (2015)
32	Lonicin E	Chen et al. (2015)
33	Ursonic acid	Yang (2019)
34	Cauloside C	Chen et al. (2015)
35	Ursolic acid	Chen et al. (2015)

### Iridoids

Iridoids have basic cores such as cyclic allyl ethers and alcoholic hydroxyl groups. Since alcoholic hydroxyl groups are hemiacetal hydroxyl groups and have active properties, such compounds mostly exist in the form of iridoid glycosides. The iridoids in LJF include common iridoid glycosides, iridoid glycosides, 4-position unsubstituted iridoid glycosides and so on. It has the pharmacological effects of protecting liver and gallbladder, anti-inflammation and analgesia, relieving pain, anti-tumor, anti-oxidation, antibacterial and so on. At present, about 66 iridoids have been isolated from LJF, which are shown in Table 5 (Song et al., 2014; Chen et al., 2015; Liu et al., 2018; Tan et al., 2018; Wu et al., 2019). The structures of iridoids are shown in Figure 5.

**TABLE 5:** Iridoids in LJF.

No.	Components	References
1	(E)aldosecologanin	Tan et al. (2018)
2	Adinoside A	Chen et al. (2015)
3	7-epimacin	Liu et al. (2018)
4	7-epiproside	Chen et al. (2015)
5	7-epilog	Liu et al. (2018)
6	8-epimacin	Liu et al. (2018)
7	8-epimalic acid	Tan et al. (2018)
8	8-epiproside	Tan et al. (2018)
9	8-epigenin	Chen et al. (2015)
10	8-Epipanic acid	Tan et al. (2018)
11	L-phenylalaninosecologanin B	Liu et al. (2018)
12	L-phenylalaninosecologanin C	Liu et al. (2018)
13	Dioxoacetin dibutyl acetal	Chen et al. (2015)
14	Dimethyl secologanoside	Song et al. (2014)
15	Demethylsecologanol-7-O-arabinoside	Wu et al. (2019)
16	Dehydromorronisi	Liu et al. (2018)
17	7-o-butyl cyclosporine	Chen et al. (2015)
18	Sweroside	Tan et al. (2018)
19	Dimethoxy-clavulazone	Liu et al. (2018)
20	7-epi-vogeloside	Tan et al. (2018)
21	7-O-ethyl sweroside-7-methyl ester	Song et al. (2014)
22	Grandifloroside	Chen et al. (2015)
23	7-O-(4-β-D-glucopyranosyloxy-3-methoxy benzoyl) secologanolic acid	Chen et al. (2015)
24	6′-O-(7α-hydroxyswerosyloxy)loganin	Chen et al. (2015)
25	Japonicaside E	Wu et al. (2019)
26	Aglycin	Tan et al. (2018)
27	Honeysuckle A-W	Chen et al. (2015)
28	Split ring mackinin	Liu et al. (2018)
29	Cleft cyclic maleic acid	Chen et al. (2015)
30	Cyclosporin	Liu et al. (2018)
31	Split epoxide	Liu et al. (2018)
32	Cyclosporin dimethyl acetal	Chen et al. (2015)
33	7-epi-schizolide hemiacetal lactone	Chen et al. (2015)
34	7-Ethyl-epi-schitolide hemiacetal lactone	Chen et al. (2015)
35	Lonjaponspiroside A	Wu et al. (2019)
36	Lonjaponspiroside B	Wu et al. (2019)
37	Lonjaponspiroside C	Chen et al. (2015)
38	Lonjaponspiroside D	Chen et al. (2015)
39	Loniphenyruviridosidea-D	Chen et al. (2015)
40	Lonijaposide A ∼ W	Chen et al. (2015)
41	Malic acid	Tan et al. (2018)
42	Loganin	Tan et al. (2018)
43	Macassin-7-one	Tan et al. (2018)
44	Dehydromononoside	Song et al. (2014)
45	7α-Monoside	Tan et al. (2018)
46	7β-Monoside	Tan et al. (2018)
47	L-phenylalaninosecologanin	Chen et al. (2015)
48	Deoxyoprolinoyiloganin A	Liu et al. (2018)
49	(1S, 6R) -8-hydroxyabscisic acid-β-D-glucoside	Chen et al. (2015)
50	Lonicoside	Chen et al. (2015)
51	Loniceracetalide A	Chen et al. (2015)
52	Loniceracetalide B	Chen et al. (2015)
53	Secoxyloganin 7-butylester	Chen et al. (2015)
54	Secosesquside-7-methylester	Chen et al. (2015)
55	Secosesquside	Wu et al. (2019)
56	Secologanic acid	Tan et al. (2018)
57	Stryspinoside	Chen et al. (2015)
58	Abscisic acid	Chen et al. (2015)
59	Urceolide	Chen et al. (2015)
60	Vogeloside	Tan et al. (2018)
61	Vaginin	Liu et al. (2018)
62	Spiroside A	Liu et al. (2018)
63	7-O-ethyl swertiain	Chen et al. (2015)
64	6′-O-Acetyl arbutin hemiacetal lactone	Wu et al. (2019)
65	6′-O-Acetyl Split-epoxidized Macanin	Wu et al. (2019)
66	7- O-ethoxymononoside	Tan et al. (2018)

### Essential oils

Essential oils are a kind of aromatic oily liquids in plants that can be distilled with steam. They mainly contain aliphatic compounds, aromatic compounds, sulfur-containing nitrogen compounds, terpenes and oxygen-containing derivatives. Most of them have the functions of expectorant and antitussive, invigorating stomach, antipyretic and analgesic, antibacterial and anti-inflammatory. At present, more than 279 essential oils have been isolated and identified from LJF, as shown in Table 6 (Wang et al., 1992; Wang Y. et al., 2008; Xia et al., 2012; Xiao et al., 2013; Chen et al., 2015; Wang, 2015; Li et al., 2017; Xia et al., 2017; Liu et al., 2018). The main structures of essential oils are shown in Figure 6.

**TABLE 6:** Essential oils components in LJF.

N0.	Components	References
1	Beta-cineol	Xia et al. (2012)
2	Patchouli alcohol	Wang et al. (2008b)
3	Methyl anthranilate	Wang et al. (2008b)
4	1,8-eucalyptol	Xia et al. (2012)
5	2-cyclohexyl acrylate	Wang et al. (1992)
6	Alpha-cedrol	Xia et al. (2012)
7	Alpha-cadinene	Xiao et al. (2013)
8	Beta-cadinene	Wang et al. (1992)
9	Delta-cadinene	Xia et al. (2012)
10	Benzaldehyde	Xiao et al. (2013)
11	Benzyl benzoate	Wang et al. (1992)
12	Ethyl benzoate	Wang et al. (1992)
13	Hexyl benzoate	Wang et al. (2008b)
14	(Z) -benzoic acid-3-hexene-1-ol ester	Wang et al. (2008b)
15	2- (benzylidene) octanol	Wang et al. (2008b)
16	Phenethyl alcohol	Wang (2015)
17	Phenylacetaldehyde	Wang et al. (2008b)
18	2-phenyl-2,4-octadienol	Wang et al. (2008b)
19	Alpha calacorene	Wang et al. (2008b)
20	Sesquiphellandrene	Xia et al. (2012)
21	Menthol	Xia et al. (2012)
22	[1ar- (1aΑ, 4Α, 4aΒ, 7bΑ)] -Octahydro-1,1,4,7-Tetramethyl-Cyclopropane [E] Pyrazine	Wang et al. (2008b)
23	1a, 2,3,3a, 4,5,6,7b-Octahydro-1,1,3a, 7-Tetramethyl-1-Hydro-Cyclopropane [A] Naphthalene	Wang et al. (2008b)
24	[1ar- (1aΑ, 7Α, 7aΑ, 7bΑ)]-1a, 2,3,5,6,7,7a, 7b-Hydrogen -1,1,7,7a-Tetramethyl-1hydro-Cyclopropane Naphthalene	Wang et al. (2008b)
25	[1R- (1Α, 3aΒ, 4Α, 7Β)]-1,2,3a, 4,5,6,7,8-Octahydro-1,4-Dimethyl-7- (1-Methylethylene Ki) O	Wang et al. (2008b)
26	[1s- (1α, 4α, 7α)]-1,2,3,4,5,6,7,8-octahydro-1,4-dimethyl-7- (1-methylethylene) o	Wang et al. (2008b)
27	(+)-Longifolene	Xia et al. (2012)
28	Citral	Wang et al. (1992)
29	Nerol	Xia et al. (2012)
30	Nerol ethyl ester	Xia et al. (2012)
31	Nerolidol	Xia et al. (2012)
32	Guaiol	Xia et al. (2012)
33	Atractylodin	Xia et al. (2012)
34	(Z)-beta-damascone	Wang et al. (2008b)
35	Eugenol	Wang et al. (1992)
36	Beta-clovene	Xia et al. (2012)
37	Beta-carotene oxygenase	Xia et al. (2012)
38	P-xylene	Liu et al. (2018)
39	Delta- cedrene	Xiao et al. (2013)
40	Anthracene	Wang et al. (2008b)
41	3,3′-dimethylbiphenyl	Wang et al. (2008b)
42	Dibenzothiophene	Wang et al. (2008b)
43	1,3-dimethylnaphthalene	Wang et al. (2008b)
44	1,4-dimethylnaphthalene	Wang et al. (2008b)
45	1,6-dimethylnaphthalene	Wang et al. (2008b)
46	1- (1,4-dimethyl-3-cyclohexen-1-yl) ethanone	Wang et al. (2008b)
47	1,6-Dimethyl-4- (1-Methylethyl)-1,2,3,4,4a, 7-hexahydronaphthalene	Wang et al. (2008b)
48	2,7-dimethylnaphthalene	Wang et al. (2008b)
49	2,2-dimethylcycloethanol	Wang et al. (1992)
50	2,4-dimethylbenzaldehyde	Wang et al. (2008b)
51	2,6-lutidine	Liu et al. (2018)
52	2,6-dimethyl-5,7-octadien-2-ol	Wang et al. (2008b)
53	2,7-dimethyl-2,6-octadien-1-ol	Wang et al. (2008b)
54	3,7-dimethyl-1,6-octadienol	Xia et al. (2012)
55	3,7-dimethyl-6-octenol	Xia et al. (2012)
56	(Z) -3,7-dimethyl-2,6-octylene-1-ol	Wang et al. (2008b)
57	3,7-dimethyl-1,5,7-octatrien-3-ol	Wang et al. (2008b)
58	4,7-Dimethyl-1- (1-Methylethyl) -1,2,3,5,6,8a-Hexahydronaphthalene	Wang et al. (2008b)
59	4a, 8-Dimethyl-2- (1-Methylvinyl) -1, 2, 3, 4, 4a, 5, 6, 8a-Octahydronaphthalene	Wang et al. (2008b)
60	4,8a -Dimethyl-6-Isopropenyl-1,2,3,5,6,7,8,8a-Octahydronaphthalen-2-Ol	Wang et al. (2008b)
61	(1s -cis) -4,7-dimethyl-1- (1-methylethyl) -1,2,3,5,6,8-hexahydronaphthalene	Wang et al. (2008b)
62	6,10-dimethyl-2-undecone	Wang et al. (2008b)
63	6,10-dimethyl-3,5,9-undecytrien-2-one	Wang et al. (2008b)
64	Trans-2- (2-pentenyl) furan	Wang et al. (2008b)
65	2,6-di-tert-butyl-4-methylphenol	Wang et al. (1992)
66	Carveol	Chen et al. (2015)
67	2,3-dihydrofarnesol	Xia et al. (2012)
68	Methyl arachidate	Wang et al. (2008b)
69	Methyl behenate	Xiao et al. (2013)
70	Methyl tetracarbonate	Chen et al. (2015)
71	N-pentacosane	Chen et al. (2015)
72	Hexacosane	Xiao et al. (2013)
73	Linalool	Liu et al. (2018)
74	Trans-2-methyl-2-vinyl-5- (α-hydroxyisopropyl) tetrahydrofuran	Xia et al. (2012)
75	Trans-linalool oxide	Xia et al. (2012)
76	Trans-farnesene	Xia et al. (2012)
77	Trans-nerolidol	Wang et al. (2008b)
78	Phenanthrene	Wang et al. (2008b)
79	Decane	Wang et al. (2008b)
80	1-decyne	Wang et al. (1992)
81	(E) 2-decenal	Wang et al. (2008b)
82	(E, e) -2,4-decadienal	Wang et al. (2008b)
83	2-heptanone	Liu et al. (2018)
84	Heptanoic acid	Wang et al. (2008b)
85	(Z) -2-heptenal	Wang et al. (2008b)
86	Trans-3-nonen-2-one	Wang et al. (2008b)
87	Germacrene-D	Xia et al. (2012)
88	Beta-rutene	Xia et al. (2012)
89	Cyclooctanol	Wang et al. (2008b)
90	Piperene	Wang et al. (2008b)
91	Βeta-bisabolene	Xiao et al. (2013)
92	Cyclopentyl methyl cyclohexane	Chen et al. (2015)
93	Cyclohexyl methyl benzene	Chen et al. (2015)
94	Cyclohexyl isobutyl oxalate	Chen et al. (2015)
95	Cyclohexyl formate	Wang et al. (1992)
96	Hexanoic acid	Wang et al. (2008b)
97	(E) -2-hexenal	Wang et al. (2008b)
98	3-hexenol	Wang et al. (1992)
99	(Z) -3-hexene-1-ol	Wang et al. (2008b)
100	Jujutrienone	Wang et al. (2008b)
101	Alpha-curcumene	Xia et al. (2017)
102	Alpha-farnesene	Xia et al. (2012)
103	Farnesyl acetone	Wang (2015)
104	Farnesol acetate	Xia et al. (2012)
105	10-dodecatrienal	Xia et al. (2012)
106	Farnesol	Wang et al. (1992)
107	1-methylphenanthrene	Wang et al. (2008b)
108	1-methylethyl 4-methyl-3-cyclopentenol	Wang et al. (2008b)
109	1- (3-methylphenyl) ethanone	Wang et al. (2008b)
110	1-methylnaphthalene	Wang et al. (2008b)
111	1-methoxy-4- (1-propenyl) benzene	Wang et al. (2008b)
112	1-methylethyl-2-hydroxybenzoate	Wang et al. (2008b)
113	1- (1-Methylethyl) -4,7-Dimethyl-1,2,4a, 5,6,8a-Hexahydronaphthalene	Wang et al. (2008b)
114	1-methyl-5-methylene-8- [1-methylethyl] -1,6-cyclodecadiene	Wang et al. (2008b)
115	1-methyl-4- (benzoyl) benzene	Wang et al. (2008b)
116	[(E, e)]-1-methyl-5-methylene-8- (1-methylethyl) -1,6 cyclodecadiene	Xia et al. (2017)
117	2- phenanthrene	Wang et al. (2008b)
118	2-methyl-5-hepten-1-ol	Wang et al. (2008b)
119	2-methylbenzaldehyde	Wang et al. (2008b)
120	2-methoxy-4-vinylphenol	Wang et al. (2008b)
121	Methyl 2-formylbenzoate	Wang et al. (2008b)
122	3-methyl-9h-fluorene	Wang et al. (2008b)
123	3a-Methyl-6-Methylenedecylhydrocyclobutane [1,2: 3,4] Pyrcyclopentene	Wang et al. (2008b)
124	4-methylphenanthrene	Wang et al. (2008b)
125	4-methyl-3-cyclohexene-1-acetaldehyde	Wang et al. (2008b)
126	4-methyl-2- (2-methyl-1-propenyl) -tetrahydropyran	Wang et al. (2008b)
127	5-methylfurfural	Wang et al. (2008b)
128	6-methyl-5-hepten-2-one	Wang et al. (2008b)
129	Copaene	Xia et al. (2012)
130	Ascorbyl palmitate	Xia et al. (2017)
131	Carane	Xia et al. (2012)
132	2-caren-4-ol	Xia et al. (2012)
133	Delta-3-carene	Xia et al. (2012)
134	Ledol	Xia et al. (2012)
135	O-hydroxyphenylacetone	Wang et al. (2008b)
136	1,2-butyl isobutyl phthalate	Wang et al. (2008b)
137	Dibutyl phthalate	Wang et al. (2008b)
138	Lilac aldehyde c	Wang et al. (2008b)
139	elemol	Xia et al. (2012)
140	Beta-elemolic acid	Xia et al. (2012)
141	Cinene	Xia et al. (2012)
142	Naphthalene	Wang et al. (2008b)
143	Alpha-pinene	Xia et al. (2012)
144	Beta-pinene	Xia et al. (2012)
145	Palmitic acid	Xia et al. (2017)
146	Methyl myristate	Wang et al. (2008b)
147	Myristic acid	Wang et al. (2008b)
148	Nonane	Wang et al. (2008b)
149	Nonanal	Xiao et al. (2013)
150	Nonanoic acid	Wang et al. (2008b)
151	(E) -nonenal	Wang et al. (2008b)
152	Β-ginsengene	Wang et al. (2008b)
153	2,3,6-trimethylnaphthalene	Wang et al. (2008b)
154	1,6,7-trimethylnaphthalene	Wang et al. (2008b)
155	1,4,6-trimethylnaphthalene	Wang et al. (2008b)
156	1,4,5-trimethylnaphthalene	Wang et al. (2008b)
157	1,1,6-trimethyl-1,2,3,4-tetrahydronaphthalene	Wang et al. (2008b)
158	1,1,6-trimethyl-1,2-dihydronaphthalene	Wang et al. (2008b)
159	4- (2,6,6-trimethyl-1,3-cyclohexadienyl) -3-buten-2-one	Wang et al. (2008b)
160	4- (2,6,6-trimethyl-1-cyclohexenyl) -3-buten-2-one	Wang et al. (2008b)
161	6,10,14-trimethyl-5,9,13-pentadectrien-2-one	Wang et al. (2008b)
162	6,10,14-trimethyl-2-pentadecanone	Wang et al. (2008b)
163	(Z, e) -3,7,11-trimethyl-2,6,10-dodecytrien-1-ol	Xia et al. (2017)
164	[1ar- (1aΑ, 4aΒ, 7Α, 7aΒ, 7bΑ)]-Decahydro-1,1,7-Trimethyl-4-Methylene-1hydro-Cyclopropane [E] Pyrene	Wang et al. (2008b)
165	6,7,10-trihydroxy-8-octadecenoic acid	Wu et al. (2019)
166	3,3,7,11-tetramethyltricyclo [5.4.0.0 (4,11)] undec-1-ol	Wang et al. (2008b)
167	3. 5-di-tert-butyl-4-hydroxybenzaldehyde	Wang et al. (2008b)
168	Undecyal	Wang et al. (1992)
169	2-dodecyl alcohol	Wang et al. (2008b)
170	2-tridecane	Wang et al. (2008b)
171	Pentadecanoic acid	Wang et al. (2008b)
172	Methyl pentadecanoate	Wang et al. (2008b)
173	Hexadecanoic acid	Wang (2015)
174	Methyl hexadecanoate	Chen et al. (2015)
175	Ethyl hexadecanoate	Chen et al. (2015)
176	2-heptadecanone	Xiao et al. (2013)
177	Octadecaldehyde	Wang et al. (2008b)
178	Octadecane	Wang et al. (2008b)
179	Ethyl octadecadienoate	Chen et al. (2015)
180	9,12-octadecadicarboxylic acid methyl ester	Xiao et al. (2013)
181	9,12,15-octadecatrienoic acid methyl ester	Chen et al. (2015)
182	9,12,15-octadecanoic acid ethyl ester	Xiao et al. (2013)
183	Caryophyllene	Wang et al. (2008b)
184	2,5-bis (1,1-dimethylethyl) -phenol	Wang et al. (2008b)
185	Terpinene	Xia et al. (2012)
186	Umbelliferone	Wang et al. (1992)
187	Cis-2-methyl-2-vinyl-5- (α-hydroxyisopropyl) tetrahydrofuran	Xia et al. (2012)
188	Cis-3-octene-1-ol	Wang et al. (2008b)
189	Cis-linalool oxide	Xia et al. (2012)
190	Cis-transfarnesol	Xia et al. (2012)
191	Cis-jasmone	Wang et al. (2008b)
192	Benzyl salicylate	Wang et al. (2008b)
193	Βeta-terpinene	Wang et al. (1992)
194	Βeta-terpineol	Wang et al. (1992)
195	2-pentylfuran	Wang et al. (2008b)
196	(+)-Aromadendrene	Xia et al. (2012)
197	(-)-Alloaromadendrene	Liu et al. (2018)
198	Geraniol	Xiao et al. (2013)
199	Citronellol	Xia et al. (2012)
200	Geranylacetone	Wang et al. (2008b)
201	Cedarwood oil	Wang et al. (2008b)
202	Octanal	Wang et al. (1992)
203	Caprylic	Wang et al. (2008b)
204	1-octen-3-ol	Wang et al. (2008b)
205	(E, e) -2,4-octadienal	Wang et al. (2008b)
206	3-octanol	Wang et al. (2008b)
207	3,5 octadien-2-one	Wang et al. (2008b)
208	Neolongene	Wang et al. (2008b)
209	2-vinyl alcohol	Wang et al. (1992)
210	2-ethylphenol	Wang et al. (2008b)
211	3-vinylpyridine	Wang et al. (2008b)
212	3-ethyl-2-methyl-1,3-hexadiene	Wang et al. (2008b)
213	4-ethyl resorcinol	Wang et al. (2008b)
214	7-acetyl-8,9-dihydroxythymol	Wu et al. (2019)
215	Ethyl cyclohexane	Liu et al. (2018)
216	2-phenylethyl acetate	Wang et al. (2008b)
217	Geranyl acetate	Wang et al. (1992)
218	Linalyl acetate	Wang et al. (1992)
219	Benzyl acetate	Wang et al. (1992)
220	Terpine acetate	Wang et al. (1992)
221	Isobornyl acetate	Wang et al. (1992)
222	Ξ-ylangene	Xia et al. (2017)
223	Γ-ylangene	Xia et al. (2012)
224	Alpha-ylangole	Xia et al. (2012)
225	Α-ylangene	Xia et al. (2012)
226	T-ylangol	Wang et al. (2008b)
227	Iso-pinenone	Wang et al. (1992)
228	Isocinol	Wang et al. (2008b)
229	5-isopropenyl-2-methylcyclopent-1-ene formaldehyde	Wang et al. (2008b)
230	Lauric acid	Xiao et al. (2013)
231	Linolenic acid	Wang (2015)
232	Γ-linolenic acid	Wang et al. (2008b)
233	Methyl linolenate	Xia et al. (2017)
234	Linoleic acid	Xia et al. (2017)
235	Methyl linoleate	Wang et al. (2008b)
236	Ethyl linoleate	Xiao et al. (2013)
237	Methyl stearate	Wang et al. (2008b)
238	Hexyl stearate	Wang et al. (2008b)
239	Ethylene-phenylacetaldehyde	Wang et al. (2008b)
240	Folate	Xiao et al. (2013)
241	Oxyfluorene	Wang et al. (2008b)
242	Fluoranthene	Wang et al. (2008b)
243	Benzyl acid	Chen et al. (2015)
244	Methyl palmitate	Wang et al. (2008b)
245	Ethyl palmitate	Wang et al. (2008b)
246	Camphor	Wang et al. (1992)
247	N-decanoic acid	Wang et al. (2008b)
248	N-decaldehyde	Wang et al. (2008b)
249	N-dodecane	Wang et al. (2008b)
250	N-tridecane	Wang et al. (2008b)
251	N-tetradecane	Wang et al. (2008b)
252	N-pentadecane	Wang et al. (2008b)
253	N-hexadecane	Wang et al. (2008b)
254	N-heptadecane	Wang et al. (2008b)
255	N-eicosane	Wang et al. (2008b)
256	N-heneicosane	Wang et al. (2008b)
257	N-docosane	Wang et al. (2008b)
258	N-tetracosane	Wang et al. (2008b)
259	N-heptacosane	Wang et al. (2008b)
260	N-nonacosane	Xia et al. (2017)
261	Phytantriol	Xia et al. (2012)
262	Α-ionone	Wang et al. (2008b)
263	Β-ionone	Wang et al. (1992)
264	Saffron aldehyde	Wang et al. (2008b)
265	2-sec-butylphenol	Wang et al. (2008b)
266	Α-syringene	Wang et al. (2008b)
267	Methyl octoate	Li et al. (2017)
268	5-methylfuran	Li et al. (2017)
269	Trans-2,4-decadienal	Li et al. (2017)
270	9-oxononanoic acid methyl ester	Li et al. (2017)
271	2-pentadecanol	Li et al. (2017)
272	2,6,-di-tert-butyl-p-cresol	Li et al. (2017)
273	10-methyl undecanoic acid methyl ester	Li et al. (2017)
274	3,5-di-tert-butyl-4-hydroxybenzaldehyde	Li et al. (2017)
275	Heptadecene	Li et al. (2017)
276	2-methylfluorene	Li et al. (2017)
277	2,6,11-trimethyldecane	Li et al. (2017)
278	Perhydrofluorene	Li et al. (2017)
279	Bromododecane	Li et al. (2017)

### Glycoside

LJF contains a variety of glycosides, such as (-)-(e) -3,5-dimethoxyphenylpropene (+)-southern candle resin; 9-o-β-d-glucopyranoside-4-o-β-d-(6-o-benzoyl)-glucopyranoside; (-)-southern candle wood resin phenol 9-o-β-d-glucopyranoside; (-)-2-hydroxy-5-methoxybenzoic acid-2-o-β-d-(6-o-benzoyl) -glucopyranoside; (-)-4-hydroxy-3,5-dimethoxybenzoic acid-4-o-β-d-(6-o-benzoyl)-glucopyranoside; (-)-(7s,8r)-4-hydroxy-3-methoxyphenylglycerin; 9-o-β-d-[6-o-(e)-4-hydroxy-3,5-dimethoxyphenylacryloyl]-glucopyranoside and so on (Chen et al., 2015).

### Other compounds

In addition to the above ingredients, LJF also contains ginkgo alcohol, phenylalanine, stigmasterol, trans cinnamic acid, β-sitosterol-3-β-glucopyranoside-6-ethyl palmitate, daucosterol, valine, (+)-n-(3-methylbutyryl-β-d-glucosyl) nicotinic acid internal salt, (+)-n-(3-methylbutyl-2-enoyl-β-d-glucosyl) nicotinic acid internal salt, 2-methylethyl-o-methyladenosine, 5-methyluracil, 5-methylol adenosine, arginine, lonijaposidesA1, A2, A3, A4, B1, B2, tyrosine, guanosine, guanosine, guanosine (3-hydroxybenzaldehyde)-adenosine monophosphate, 4-hydroxybenzaldehyde, 6-hydroxymethyl-3-hydroxypyridine, bisresistin, abscisic acid, ursolic acid, adenosine, isoleucine, syringin, sucrose and other compounds as well as B, Ba, Ca, Cu, Co, Cr, Fe, K, Li, Mn, Mg, Mo, Ni, Pb, Sr, Ti, V, Zn and other microelement (Shang et al., 2011; Li et al., 2020).

Researchers are currently focusing on determining the organic acids, iridoids, and flavonoids in LJF. Coumarins, steroids, and polysaccharides have been studied relatively little, possibly due to their low content and complex component, which are difficult to separate and purify. LJF has a wide range of medicinal values, and its annual use is particularly large, so the annual production of LJF is in short supply. Studies have shown that the flavonoid content in the leaves of LJF is two times that in the flowers, and the polysaccharide content in the stems is 1.5 times that in the flowers. On the other hand, a large number of LJF stems and leaves were discarded, which caused both resource waste and environmental pollution. Polysaccharides in LJF have been shown to have immunomodulatory and antiviral properties in pharmacological studies. Compared with LJF buds, the collection of stems and leaves was easier and the price was lower. These leaves and leaves, especially the leaves of LJF, should be fully utilized.

## Pharmacological effects

### Antipyretic effect

LJF has the function of clearing heat and detoxicating since ancient times, and its heat-clearing mechanism has been extensively studied in modern pharmacology. Duan Hongyan et al. found that the antipyretic, anti-free radical injury and immune enhancement of LJF on febrile rats were related to the increase of nitric oxide (NO) and interleukin-6 (IL-6) content *in vivo* (Duan and Cheng, 2009). However, the study lacked a positive drug group. Song Jianhua et al. studied the antipyretic and anti-inflammatory effects of LJF on yeast-induced fever model mice and xylene-induced inflammatory model mice. It was found that all dose groups of LJF had obvious antipyretic and anti-inflammatory effects in a dose-dependent manner (*p* < 0.05) (Song, 2011). Wang Yaqiong et al. studied the antipyretic mechanism of LJF. The results showed that the antipyretic and detoxifying effect of LJF was related to the inhibition of tricarboxylic acid cycle metabolic pathway in rats and the decrease of succinic acid, α-ketoglutaric acid and malic acid. it is related to the increase of tricarboxylic acid intermediates such as 3- hydroxybutyric acid, leucine and isoleucine (Wang Y. et al., 2016). Li Xingping et al. studied the antipyretic effects of chlorogenic acid and luteolin on animal febrile models induced by 2Magna 4-dinitrophenol, yeast and endotoxin. The results showed that different doses of LJF extract had good antipyretic effect on the above-mentioned febrile model, but chlorogenic acid and luteolin had no obvious antipyretic effect (Li et al., 2012).

Peng sha et al. established a system dynamics model and found that the efficacy of LJF in heat-clearing and detoxicating was related to the inhibition of the expressions of interleukin-1 (IL-1) and Jun N-terminal kinase (JNK) in IL-1 signaling pathway and the expression of IL-6 and C-reactive protein (CRP) in IL-6 signaling pathway. At the same time, it was further found that chlorogenic acid and linalool were involved in the regulation of IL-1 signaling pathway, while chlorogenic acid and luteolin were involved in the regulation of IL-6 signaling pathway (Peng et al., 2020). This study provided the basis for the active components and action mechanism of heat-clearing and detoxicating of LJF. However, computer simulation alone cannot improve enough clinical basis, so it is recommended that the further experiments should be verified *in vivo* and *in vitro*.

LJF has antipyretic and analgesic effects in a dose-dependent manner *in vivo*. Such effects may be related to the regulation of the expressions of IL-1, Jun, IL-6 and CRP, and the regulation of tricarboxylic acid cycle pathway, IL-1 signaling pathway and IL-6 signaling pathway. However, these experiments have only studied the efficacy or detected the expression of only a few proteins in the pathway, and the research is not in-depth. These conclusions are not enough to prove the entire mechanism of LJF antipyresis.

### Anti-inflammatory effect

The antipyretic effect of LJF is closely related to its anti-inflammatory effect, and it is called “traditional Chinese medicine antibiotic”. Yan Xuelong et al. found that LJF exerts its anti-inflammatory effect by inhibiting pro-inflammatory cytokines such as tumor necrosis factor alpha (TNF-α), IL-1β and IL-6, regulating the balance of TH1/TH2, increasing cytokines secreted by TH1 cells or reducing cytokines secreted by TH2 cells (Yan et al., 2016). However, its material basis and mechanism of action are not clear. Song Yaling et al. investigated the effects of LJF on NO, TNF-α and IL-6 produced by macrophage RAW264.7 cells stimulated by lipopolysaccharide (LPS), and studied the anti-inflammatory activity of phenolic acids in LJF *in vitro*. It was found that caffeic acid, methyl caffeate, chlorogenic acid, neo-chlorogenic acid, crypto-chlorogenic acid, iso-chlorogenic acid A, iso-chlorogenic acid B and iso-chlorogenic acid C could inhibit the inflammatory cytokines produced by RAW264.7 cells stimulated by LPS in different degrees, among which caffeic acid had the strongest inhibitory activity (Song et al., 2015). However, the study lacked a positive drug group. Miao Yanyan et al. established the fingerprint of LJF and studied the spectrum-activity relationship of its anti-inflammatory action. The results showed that 17 compounds had close positive correlation with the anti-inflammatory efficacy (Miao et al., 2020).

Li Kangning et al. used LPS-induced acute anterior uveitis (AAU) mice to explore the anti-inflammatory effect of LJF and clarify its mechanism of action. The results showed that the inflammatory cell infiltration in uveal tissue was reduced to different degrees in the low, medium and high doses of LJF extract group. The mRNA and protein expression levels of Toll-like receptor 4 (TLR4) and nuclear transcription factor-κB (NF-κB) in the eye tissue of mice in the LJF extract group were significantly reduced, while the levels of TNF-α, IL-6 and IL-10 were significantly reduced. LJF extract down-regulated the expression of inflammatory cells through the TLR4/NF-κB signaling pathway to reduce the inflammatory response in the eye tissue of AAU mice, in a dose-dependent manner (Li et al., 2021).

Lou et al. studied the mechanism of chlorogenic acid on rheumatoid arthritis. The results showed that chlorogenic acid could reduce the expression of p-signal transducer and activator of transcription 3 (p-STAT3) and Janus kinase 1 (JAK1) in the JAK/STAT pathway and the expressions of p50 and IKK in the NF-κB pathway, and inhibit the inflammatory proliferation of fibroblast like synoviocytes caused by IL-6, thereby exerting the anti-inflammatory effect (Lou et al., 2016). Kao et al. studied the therapeutic effect of LJF 50% ethanol extract on LPS-induced acute lung inflammation mice. Studies had found that LJF could downregulate the expression of TNF-α, IL-6 and inducible nitric oxide synthase (iNOS), and upregulate the expression of IL-10. At the same time, FLJ also inhibited the expressions of NF-κB, STAT3, IκB, JNK and p38 phosphorylation, and increased the phosphorylation of Sp1 to produce an effective anti-inflammatory effect on the lung (Kao et al., 2015).

LJF is effective for a variety of diseases, such as acute anterior uveitis, rheumatoid arthritis, and acute lung inflammation, caused by inflammation. The anti-inflammatory mechanism of LJF has been studied primarily at the *in vitro* and *in vivo* levels. At present, it has been found that LJF can inhibit the expression of inflammatory factors such as TNF-α, IL-6 and iNOS, and regulate TLR4/NF-κB signaling pathway and JAK/STAT pathway, thus playing an anti-inflammatory role. Organic acids are the main anti-inflammatory components. However, anti-inflammatory components and mechanism have not been adequately researched. Future scholars can expand their scope to explore other components of anti-inflammation in LJF, such as saponins and essential oils.

### Antimicrobial effect

LJF has broad-spectrum antibacterial effect, which has been verified by many pharmacological experiments *in vitro* and *in vivo*. Feng Xiuli et al. used agar diffusion method to study the *in vitro* antibacterial activity of LJF against *Escherichia Coli, Candida Albicans, Bacillus Subtilis, Aspergillus Niger* and *Staphylococcus Aureus*. The results showed that all the five kinds of bacteria were sensitive to the water extract of LJF (Feng et al., 2013). However, only a single concentration of water extract of LJF and the lack of positive drug group are the two limitations of this experiment.

Hu Xuan et al. found that the aqueous extract of LJF has bacteriostatic and bactericidal effects on *escherichia coli, candida albicans, klebsiella pneumoniae, staphylococcus aureus, pseudomonas aeruginosa and streptococcus B* (Hu et al., 2015a). Great efforts have been made by the researchers in the search for the antibacterial active ingredient of LJF. (Han et al., 2014) conducted a comparative study of the antibacterial effects of 3, 4-di-O-caffeoylquinic acid (3, 4-diCQA), 3, 5-di-O-caffeoylquinic acid (3, 5-diCQA), and 4,5-di-O-caffeoylquinic acid (4, 5-diCQA) on *Bacillus shigae*. The order of efficacy was determined to be 3, 5-diCQA > 4, 5-diCQA > 3, 4-diCQA. by comparing the IC50 values of each compound. This experiment has shown that slight differences in chemical structures of similar compounds can also greatly affect their drug efficacy. In this experiment, the changes of the position and distance of caffeyl ester groups greatly affected the antibacterial effect (Han et al., 2014). Yang et al. studied the antibacterial activity of 7-acetyl-8,9-dihydroxy thymol, first isolated from LJF, and the known 7,8-dihydroxy-9-buyryl thymol. Two compounds were found to have antibacterial activity against *Staphylococcus aureus, Escherichia coli, Micrococcus luteus, and Bacillus cereus*. IC50 values range from 27.64 ± 2.26 to 128.58 ± 13.26 μg/ml (Yang et al., 2018). Wang Qing et al. showed that LJF had good inhibitory effect on standard *staphylococcus aureus, staphylococcus epidermidis, escherichia coli, diplococcus pneumoniae, staphylococcus aureus, bacillus subtilis, staphylococcus aureus, pseudomonas cepacia and streptococcus B*. The minimum inhibitory concentration was 37.5–100.00 mg. Different concentrations of LJF extract could also reduce the mortality of mice caused by *diplococcus pneumoniae and staphylococcus aureus* and prolong the survival time of infected mice (Wang Q. et al., 2008). Guan Zhongying et al. found that LJF extract could well inhibit *streptococcus pneumoniae and pseudomonas aeruginosa*, the diameter of bacteriostatic zone was more than 19mm, and the minimum inhibitory concentration of LJF extract to *escherichia coli, streptococcus pyogenes, staphylococcus aureus, shigella dysenteriae and streptococcus agalactis* was less than 0.125 g/ml (Guan et al., 2009). However, the above studies are lack of research on the basis and mechanism of anti-bacterial substances of LJF.

Zhang Zhongbin et al. found that phenolic acids in LJF were positively correlated with antibacterial activity (Zhang et al., 2019). Future scholars can use this as a reference to conduct in-depth study on the antibacterial material basis and mechanism of LJF, so as to provide a scientific basis for clinical application. Zeng Huaqian et al. determined the minimum inhibitory concentration of LJF on *Streptococcus mutans* UA159 by liquid double dilution method. LJF could inhibit the growth, acid production and adhesion of *Streptococcus mutans* UA159 and reduce the formation of biofilm (Zeng et al., 2022). Shi et al. through the construction of spectrum-activity relationship found that LJF had an antibacterial effect against *P.aeruginosa*, a common strain in wound sites, and chlorogenic acid and 3,4-dicaffeoylquinic acid were the main components of LJF that had antibacterial effect through the construction of spectrum-activity relationship (Shi et al., 2016). Yang et al. studied the antibacterial activity of five kinds of iridoid glycosides firstly isolated from LJF against *Staphylococcus aureus* ATCC 25923. The results showed that all the compounds showed slight inhibitory activity, with MIC values ranging from 13.7 to 26.0 g/ml (Yang et al., 2019).

The above studies have found that LJF extract has a significant antibacterial effect, and the effective components include phenolic acids, thymol derivatives and iridoid glycosides. These experimental results further enriched our understanding of the antibacterial activity of LJF and provided a scientific basis for the clinical treatment of drug-resistant pathogens. However, the antibacterial mechanism has not been studied in depth, and the mechanism by which these compounds inhibit the synthesis of bacterial cell walls or interfere with the synthesis of protein or inhibit the transcription and replication of nucleic acids has not been elucidated. In addition, most studies did not set up a positive group, and did not study the targets and pathways of compound action. LJF has a broader antibacterial spectrum than other common antibacterial agents. Further studies should also be conducted on the antibacterial components in LJF, such as polysaccharides and essential oils. At the same time, most bacteria develop drug resistance due to the abuse of antibiotics, and further research should be conducted on the efficacy of LJF against drug-resistant bacteria.

### Antiviral effect

Mi Huijuan et al. established the antiviral spectral effect model of LJF and found that the IC50 of 10 batches of LJF was between 0.911 and 2.441 g/L (Mi et al., 2015). Wang Bianli et al. studied LJF and its antiviral activity by spectrum-effect relationship, and found that LJF had significant inhibitory activity on influenza A virus H1N1, and the treatment index of 80 batches of LJF against influenza A virus H1N1 was 14.57–39.85, which in some batches was higher than that of positive control drug ribavirin (16.78). However, it had no significant effect on herpes simplex virus type I and hand-foot-mouth disease virus EV71 strain in the LJF (Wang et al., 2015).

Li Meiyu et al. observed the inhibitory effect of LJF on human respiratory syncytial virus type 3 in human cervical cancer cell line (Hela). It was found that LJF could directly inactivate Hela cells (IC50 = 0.16 mg/ml, TI = 31.2), and LJF could also inhibit respiratory syncytial virus biosynthesis (IC50 = 1.0 mg/ml, TI = 5). The results showed that LJF had antiviral effect *in vitro* mainly through direct inactivation, inhibition of virus adsorption and inhibition of biosynthesis (Li, 2010). The experiments of Liu Ying et al. *in vitro* showed that the extract of LJF had obvious antiviral effect on vero cells infected with herpes simplex virus type I. the maximum non-toxic concentration was 384 mg/L and the therapeutic index was 26.56. The results of *in vivo* experiments showed that LJF had a good therapeutic effect on herpes simplex keratitis, which could reduce the degree of keratopathy and shorten the cure time (Liu and Wang, 2011). However, the above studies are lack of research on the active material basis of LJF. Ma Shuangcheng et al. screened the anti-respiratory virus components in LJF, and finally determined that luteolin-7-O-glucoside and luteolin were the active components of LJF with strong anti-respiratory virus (Ma et al., 2006). Chen Juanjuan et al. found that chlorogenic acid in LJF has a good anti-HCMV effect. The maximum non-toxic concentration was 100 μg/ml, the minimum effective concentration was 1 μg/ml, and the therapeutic index was 100 (Chen et al., 2009).

Ding Jie et al. established the fingerprint of LJF polysaccharide and investigated its inhibition effect on respiratory syncytial virus (RSV). The results showed that there were 12 common peaks in the GC fingerprint of 12 batches of LJF medicinal materials with the similarity ≥ 0.994. The EC50 values of the total polysaccharide, 80% alcohol-precipitated polysaccharide, 50% alcohol-precipitated polysaccharide and 20% alcohol-precipitated polysaccharide of LJF (S12) were 0.76, 0.61, 1.03 and 3.04 g/L, respectively. The TI values were 15.36, 18.51, 11.69 and 4.22, respectively. The results showed that LJF had certain RSV inhibitory activity *in vitro* (Ding et al., 2020).

In addition, LJF also has effects in anti-avian influenza virus, anti-coxsackie virus agraine B, anti-Ecco virus, anti-rubella virus, anti-poliovirus, anti-varicella zoster virus, anti-adenovirus, anti-porcine reproductie and respiratousyndrome virus, anti-pseudorabies virus, anti-human immunodeficiency virus and other effects (Zhang et al., 2016). Kashiwada et al. studied the antiviral activity of six secoridoids compounds in LJF against influenza A virus. Studies have shown that vogelosine, 7-epi-Vogelosine, secoxyloganin, dimethyl secologanoside and sweroside can inhibit the virus at 100 μg/ml, and the inhibition range is 53.1–28.4%. Among them, secoxyloganin showed the best inhibition effect, reaching 53.1% (Kashiwada et al., 2013).

To sum up, the current research shows that LJF has good antibacterial and antiviral effect. The antiviral components in LJF are flavonoids, polysaccharides and secoiridoid glucosides. As a medicinal material with homology of medicine and food, it is expected to become an important screening target of new antibacterial and antiviral drugs because of its natural properties, low-toxic and high in efficacy, as well as broad spectrum antibacterial properties and heat-clearing and detoxification effects. The symptoms of novel coronavirus (COVID-19) that broke out in 2019 are generally fever, cough and dyspnea. With Lianhua Qingwen capsule, a Chinese medicine preparation containing LJF, playing a preventive and therapeutic intervention in COVID-19 patients. Traditional Chinese medicine, particularly LJF, has demonstrated that it can significantly reduce fevers and improve breathing in COVID-19 patients by relieving their symptoms (Luo et al., 2021; Yao et al., 2022).

### Antioxidation

Liu Hao et al. analyzed the antioxidant activity of ethanol extracts of LJF with different concentrations. The results showed that 95% ethanol LJF extract had the strongest scavenging effects on OH and DPPH, with the scavenging rate of 90.69% and 65.64%, respectively. Further studies showed that the content of chlorogenic acid, flavonoids and polyphenols in the 95% ethanol extract was also the highest, indicating that the content of these compounds was positively correlated with antioxidant activity (Liu et al., 2016). Tian Lei et al. found that different proportions of LJF extract had good inhibitory effects on DPPH, OH and superoxide anion radical (O2-[O]), increased the content of NO in the supernatant of damaged HUVECs cells, decreased the release of lactate dehydrogenase (LDH), and played a good role in anti-free radicals and antioxidation (Tian et al., 2014). Xu Xiaobo et al. compared the antioxidant activity of extracts from the stems, leaves and flowers of LJF. The results showed that when the concentration of flower extract was 0.22 mg/ml and 0.18 mg/ml, the DPPH free radical and ABTS + free radical scavenging rates were the highest among the three, reaching 90% and 90%, respectively. The IC50 values for DPPH radical scavenging and ABTS +·radical scavenging of the flower, stem and leaf extracts were 0.07, 0.13, 0.17 and 0.05, 0.10, 0.15 mg/ml, respectively. Chlorogenic acid and caffeic acid were also found to have the highest contents in the flower extracts (Xu et al., 2018). Liu Changping et al. also found that flavonoids in LJF can block the autoxidation of linoleic acid and lard (Liu, 2009). The antioxidant activity of LJF was studied from different parts, extraction methods and doses of LJF in the above experiments, and good antioxidant activity of LJF *in vitro* was obtained. However, drugs should eventually be administered *in vivo*, which is different from a single *in vitro* environment and contains many cytokines. The results of antioxidant experiments *in vitro* may not be achieved *in vivo*. Therefore, the antioxidant effect and mechanism of LJF in animals should be further studied.

Luo Lei et al. found that different doses of LJF flavonoids could significantly promote the proliferation of RAW264.7 cells, increase the total antioxidant capacity and the activities of catalase (CAT), glutathione peroxidase (GSH-px) and superoxide dismutase (SOD), decrease the activity of LDH and malondialdehyde (MDA) content, and protect RAW264.7 cells from hydrogen peroxide-induced injury in a dose-dependent manner (Luo et al., 2018). Liang Conglian et al. established the spectral effect model of scavenging DPPH free radicals in LJF, in order to find the active components with scavenging DPPH free radicals in LJF. The results showed that there was a significant negative correlation between the contents of isochlorogenic acid B and isochlorogenic acid C and the scavenging activity of DPPH free radicals (Liang et al., 2015). Li Ying et al. found that methyl 3,4-dicaffeoylquinic acid (IC50 = 8.36 μmol/L), quercetin (IC50 = 6.46 μmol/L) and luteolin (IC50 = 2.08 μmol/L) in LJF had good xanthine oxidase inhibitory activity. Among them, the antioxidant activity of flavonoid aglycone > flavonoid glycosides, dicaffeoylquinic acid methyl ester > dicaffeoylquinic acid > mono-caffeoylquinic acid, and the 5-position substitution of dicaffeoylquinic acid made the greatest contribution to the antioxidant activity of the compounds (Li et al., 2011). This provides a scientific basis for the preparation of antioxidants from LJF.

Zhao Minmin et al. established an offline two dimensional-High Performance Liquid Chromatography-DPPH-Electrospray ionization-Quadrupole-Time of Flight/mass (2D-HPLC-DPPH-ESI-Q-TOF/MS) technique to screen the antioxidant components in LJF. The results showed that a total of 36 components with antioxidant activity were screened out, and seven components including isochlorogenic acid B, isochlorogenic acid A, 1,3-dicaffeoylquinic acid, isochlorogenic acid C, rutin, cryptochlorogenic acid, and chlorogenic acid were verified to have good antioxidant activity *in vitro* (Zhao et al., 2020). Lv Rui et al. applied HPLC to determine the contents of multiple organic acids, luteolin-glucoside and anthocyanin components in LJF at different florescence and the relationship between functional components and antioxidant activity during different florescence. The results showed that there was a significant positive correlation between the content of chlorogenic acid and DPPH and ABTS + clearance, and there was a significant positive correlation between the content of neochlorogenic acid, crypto-chlorogenic acid and luteolin and ABTS + clearance. Florescence was negatively correlated with anthocyanin content and DPPH clearance (Lv and Xu, 2022).

Xiao et al. studied the antioxidant effects of 4,5-di-O-caffeoylquinic acid methylester (4,5-CQME) isolated from LJF on the liver oxidative damage HepG2 cells model induced by H2O2. They found that 4,5-CQME could up-regulate the mRNA and protein expressions of NAD (P)H:quinone oxidoreductase 1 (NQO1), and HO-1 and down-regulate the expression of Kelch-like ECH-associated protein 1 (Keap1) and promote nuclear factor erythroid-2 related factor 2 (Nrf2) translocation, thus inhibiting oxidative stress by regulating Keap1/Nrf2 pathway. While the Nrf2 inhibitor ML385 can reduce the protection of 4,5-CQME (Xiao et al., 2020). Han et al. studied the antioxidant effect of chlorogenic acid on osteoblast MC3T3-E1 cell oxidative stress model induced by H2O2. Chlorogenic acid was found to significantly reduce H2O2-induced oxidative damage and activate the phosphorylation of Protein kinase B (Akt) by up-regulating the protein expression of HO-1 and the upstream mediator Nrf2. After administration of LY294002(Phosphoinositide 3-kinase (PI3K)/Akt inhibitor), the expression of HO-1 and Nrf2 was inhibited again, indicating that chlorogenic acid protected cells from oxidative damage through PI3K/Akt-mediated activation of the Nrf2/HO-1 pathway (Han et al., 2017).

The main antioxidant components in the above study were organic acids and flavonoids in LJF, and most of the indicators involved were the DPPH free radicals scavenging activity. Studies have shown that the antioxidant effects of organic acids may be achieved by regulating the Keap1/Nrf2/HO-1 pathway and PI3K/Akt pathway. However, the current experiments are limited to cell-level research. Future researchers should also verify this mechanism at animal and clinical levels to study the antioxidant mechanism and ability of LJF *in vivo*. Synthetic antioxidants often have some toxic and side effects, and LJF is expected to become a low-toxicity and high-efficiency food antioxidant. Organic acids and flavonoids are also abundant in the stems and leaves of LJF. The pruning of LJF produces a large number of stems and leaves that should not be discarded as medicinal or natural antioxidant preparation.

### Immunomodulatory effect

Pi Jianhui et al. showed that flavonoids in LJF could significantly increase the organ index of immunosuppressed mice, increase the activities of acid phosphatase (ACP), alkaline phosphatase (AKP) and lysozyme (LSZ) in plasma, increase the levels of total antioxidant capacity (T-AOC) and SOD, and decrease the contents of monoamine oxidase (MAO) and MDA. It has good immunomodulatory function (Pi et al., 2015). Yin Hongmei et al. found that LJF polysaccharides had obvious immunomodulatory effect, and the efficacy was positively correlated with the dose (Yin et al., 2010). Zhou Xiuping et al. have shown that LJF can increase the phagocytic index of macrophages, enhance the transformation rate of lymphocytes, and enhance the secretion of IL-2, Interferon-γ (IFN-γ) by Thl cells. The mRNA expression of TNF-α is well developed and plays an immunomodulatory role (Zhou et al., 2011). The above experiments studied the optimal extraction process of LJF in terms of immune activity, and discussed the mechanism of LJF at the molecular level, and proved that LJF has a good immune regulatory effect at the animal level. However, the optimal dose of LJF in immune regulation still needs to be studied to lay a foundation for future practical application.

Zhang Wenwen et al. analyzed the proliferation effect of LJF on mouse lymphocytes through lymphocyte trans-formation experiment. The results showed that all the essential oils from LJF could significantly promote the transformation of mouse lymphocytes, but the pharmacological activity of the essential oil from LJF in enhancing immunity was more significant (Zhang et al., 2020). Zhou et al. studied the immunomodulatory effect of LJF polysaccharide on immunosuppressed mouse models induced by cyclophosphamide. It has been discovered that LJP can significantly increase the thymus and spleen indices of mice, facilitate the activation of spleen lymphocyte proliferation, enhance the phagocytosis of macrophages and natural killer (NK) cell activity, and enhance the immune function of mice. LJP also upregulated IL-2, TNF-α, and IFN-γ levels and increased the percentage of CD4 and CD8 T-cell subsets and the CD4/CD8 ratio. Experiments have shown that LJP can be used as a potential immunomodulator (Zhou et al., 2018). Bai et al. also performed *in vivo* studies on the immunoregulatory effects of LJF polysaccharides. The mouse model of allergic rhinitis was established by ovalbumin induction. LJF significantly inhibited the sneezing frequency and the number of nasal wipe movements, and inhibited the infiltration of inflammatory cells in the nasal mucosal tissue of model mice after administration. Biologically, LJF inhibited serum levels of immunoglobulin E (IgE), TNF-α, IL-1β, and IL-17. LJF also inhibited mRNA expression levels of IL-4, IL-5, IL-6, IL-17, IL-23, retinoic acid receptor-related orphan receptor γt (ROR-γt) and STAT3, inhibited protein expression levels of ROR-γt and STAT3, and increased mRNA and protein expression levels of Suppressor Of Cytokine Signaling 3 (SOCS3). The results showed that LJP had a therapeutic effect on allergic rhinitis by regulating the immune response of Th17 cells (Bai et al., 2020). This study provided the basis data for further application of LJP to functional foods.

In the above studies, polysaccharides, essential oils and flavonoids are the main components of LJF that play an immune regulation. Particularly, polysaccharides are the most studied and also play a major role in LJF. LJF regulates immune diseases mainly by regulating inflammatory and immune factors in immune T cells and promoting NK cell activity, and can also act on STAT3/ROR-γt pathway, which is the key part of TH17 cell differentiation. However, there is currently no unified specification for the extraction of polysaccharides from LJF, resulting in uneven polysaccharide content and lack of relevant research on the optimal and effective dose of LJF for immune regulation. This has created great difficulties for its clinical application. In the future, new preparation technologies such as microspheres, nanoparticles, and liposomes should be applied to the research and development of LJF polysaccharide preparations to improve the stability and bioavailability *in vivo*.

### Hepatoprotective effect

Li Xiansheng et al. have shown that total saponins in 70% alcohol extract of LJF at different doses can increase the activity of serum SOD, reduce the content of MDA, and inhibit hepatomegaly induced by CCl4 in mice, thus protecting the liver (Li, 2009). Liu Chang et al. used network pharmacology and experimental verification to predict the targets of LJF for the prevention of acute alcoholic liver injury and established an SD rat model of acute alcoholic liver injury. The results showed that serum aspartate aminotransferase (AST), alanine aminotransferase (ALT) and liver tissue MDA levels of rats in the high, medium and low dose groups of LJF were significantly reduced, serum IL-6 content of rats in the low and high dose groups of LJF was significantly reduced, and the SOD and GSH-Px contents of liver tissue in the medium and high dose groups of LJF were significantly increased. The PCR results showed that high, medium and low dose groups of LJF could significantly reduce the expression levels of mitogen-activated protein kinase kinase 4 (MAP2K4) and MAPK3 genes (Liu et al., 2021).

Tu Suiping et al. determined the testosterone metabolism rate of cytochrome P450 family 3 subfamily A (CYP3A) substrate in rat liver microparticles by HPLC, and determined the mRNA expression levels of CYP3A1 and CYP3A2 enzymes in rat liver by PCR technology, to investigate the effects of LJF extract on CYP3A enzyme activity and gene expression in rats. The results showed that long-term administration of LJF had no significant effect on CYP3A enzymes in rats (Tu et al., 2020). Yan Shihao et al. used a C57 mouse model of hepatic fibrosis to explore the effect of the combination of LJF and Forsythia suspensa extract on hepatic fibrosis. The results showed that after intervention with different doses of LJF-Forsythia suspensa composition, the mRNA expressions of Collagen type I alpha 1 (CollA1), Collagen alpha-1(III) chain (Col3a1), fibronectin (FN) and transforming growth factor-β (TGF-β) in the liver of model mice were significantly down-regulated, as well as the mRNA and protein expressions of alpha-smooth muscle actin (α-SMA) (Yan et al., 2021). Other studies have shown that LJF can protect the liver by reducing the levels of γ-glutamyl transpeptidase (γ-GT) and alkaline phosphatase (ALP) in serum, increasing the levels of albumin (ALB) and total protein (TP), and reducing the content of GSH-Px in liver (Teng et al., 2014; Teng et al., 2016).

Xu Xiaoyan et al. used SD rats with liver fibrosis to explore the effect and mechanism of total flavonoids of LJF on the improvement of CCl4-induced liver fibrosis. The results showed that compared with the model group, the liver fibrosis was significantly alleviated in the low-dose and high-dose groups of total flavonoids of LJF. The ALT activity in serum, hydroxyproline (Hyp) content and MDA content in liver tissue were significantly decreased, and the activities of T-SOD and GSH-Px were significantly increased. The total flavonoids of LJF can inhibit the proliferation and promote the apoptosis of hepatic stellate cells (HSC) in different degrees (Xu et al., 2020). Ge et al. studied the liver protection effects of four newly isolated flavonoids (japoflavone A-D) and ten known flavonoids on H2O2-induced in SMCC 7721 cells and HepG2 cells. It was found that japoflavone D could improve H2O2-induced apoptosis of hepatocytes and reverse the decreases of CAT and SOD in the model group in a dose-dependent manner. Japoflavone D can be used as a new hepatoprotective drug for further study (Ge et al., 2018). Tzeng et al. studied the liver protection of LJF ethanol extract on nonalcoholic steatohepatitis (NASH) model mice after induction of methionine-choline deficiency diet for 8 weeks. LJF was found to significantly improve inflammation and fibrosis in the liver of model mice, and inhibit ALT, AST in plasma and MDA levels in liver tissues. LJF also inhibited the mRNA expression of Cytochrome P450 Family 2 Subfamily E Member 1 (CYP2E1), TNF-α, TGF-β1, α-SMA, Collagen I, matrix metallopeptidase 2 (MMP2), MMP9, and diacylglycerol o-acyltransferase 2 (DGAT2) in liver tissue and up-regulated the mRNA Expression of peroxisome proliferator-activated receptor-α (PPAR-α). The liver protective effect of LJF on NASH is also related to up-regulating the expression of p-ERK and reducing the expression of p-JNK in MAPK signaling pathway (Tzeng et al., 2015).

In summary, LJF has effects in treating acute liver injury, non-alcoholic steatohepatitis, and liver fibrosis. In recent years, more and more studies have preliminarily confirmed the effect of LJF on the liver. Saponins and flavonoids are the components of LJF with hepatoprotective effect. It was found that the pathway in which LJF worked involved the extracellular regulated protein kinases (ERK) and JNK pathways in the mitogen-activated protein kinase (MAPK) signaling pathway, but had no effect on the p38 pathway. Although the research on liver protection is getting deeper and deeper, there are still some problems need to be further discussed. For example, at present, the pathways involved in liver injury also include NF-κB signaling pathway and STAT3 signaling pathway, and whether LJF also acts on these pathways needs further verification (Xu et al., 2014; Kim et al., 2020). The key components for hepatoprotective effect of LJF are mainly concentrated on LJF extract, and the mechanism of action of saponins and flavonoids in LJF extract is lack of research.

### Anti-tumor effect

Liu Yuguo et al. found that polysaccharides in different doses of LJF could regulate b-cell lymphoma-2/bcl-2-associated x (Bcl-2/Bax) apoptosis pathway; middle and high doses of LJF polysaccharides could increase the content of TNF-α in serum (*p* < 0.05) and inhibit S180 sarcoma(the inhibition rate was 23.85%, 30.02% respectively) (Liu et al., 2012). Liu Bei et al. found that LJF polysaccharides (10-250 μg/ml) had antitumor activity by promoting the proliferation of spleen lymphocytes in mice, especially at the concentration of 100 μg/ml (*p* < 0.01) (Liu and Liu, 2013). These provide a scientific reference for the further study of the anti-tumor mechanism of LJF. However, these studies lack the validation of the anti-tumor target of LJF and the optimal extraction method and dose size for its clinical application.

Lin et al. studied the anti-tumor effect of LJF polysaccharide LJ-02-1 with molecular weight of 54 kDa on pancreatic cancer cells, and they found that the inhibition rates of LJF polysaccharide on BxPC-3 and PANC-1 pancreatic cancer cells at the concentration of 1 mg/ml were 66.7% and 52.1%, respectively (Lin et al., 2016). Park et al. studied the anti-cancer effect of LJF polyphenol extract on human HepG2 cells. Polyphenol extracts were found to inhibit HepG2 cell migration in a dose-dependent manner and reduce the expression of MMP-2 proteins associated with cell migration. The polyphenol extract can prevent cell G2/M phase and induce apoptosis, inhibit the expression of mitotic-related cyclin dependent kinase 1 (CDK1), cell division cycle 25c (CDC25C), and cyclin B1 proteins, inhibit the expression of apoptosis-related proteins Bcl-xL, pro-caspasae-3, pro-caspase-9 and poly(ADP-ribose) polymerase (PARP), and promote Bax expression. The polyphenol extract can dose-dependently inhibit p-PI3K, p-Akt expression, promote p-ERK1/2, p-JNK and p-38 MAPK expression, and exert anti-tumor effect by regulating PI3K/Akt and MAPKs pathways (Park et al., 2012).

Patil et al. synthesized LJF gold nanoparticles and found that it could inhibit HeLa cells by 10.54, 29.38, 47.58 and 62.73% at 200, 300, 400 and 500 μg/ml (Patil et al., 2019). Rajivgandhi et al. synthesized LJF silver nanoparticles using the nano-silver technology and found that it effectively inhibited the viability of A549 lung cancer cells by increasing ROS production at 75 μg/ml (Rajivgandhi et al., 2022).

The above research has revealed that LJF has good anti-tumor effect on pancreatic cancer, liver cancer, cervical cancer and lung cancer, and its active components are polysaccharides and polyphenols. The anticancer effect and mechanism of LJF may be related to the regulation of PI3K/Akt and MAPKs pathways and intrinsic apoptotic pathway. In addition, LJF has been studied in recent years with respect to its nano-silver and nano-silver composites, which exhibit a good anticancer effect. However, in the above studies, no anticancer study was conducted on the single compound of LJF, and no in-depth verification of the related mechanisms was conducted in animals and clinic, which makes it difficult to ensure the clinical safety and effectiveness of LJF. In the future, researchers can study the anti-cancer effects of luteolin and chlorogenic acid, and use the popular drug nano controlled release system to study the targeted and localized administration of LJF.

### Hypoglycemic and hyperlipidemic effect

Chen Xiaolin et al. found that the water extract of LJF could reduce glucose by inhibiting the activities of α-amylase and α-glucosidase, and the inhibitory effect increased with the increase of the concentration of the extract (Chen, 2010). However, this study lacks positive drug group and control group. Ye Qinghua used streptozotocin (STZ) to induce type Ⅱ diabetic rat model. After 42 days of administration of LJF extract, it was found that the levels of blood glucose and insulin in the treatment group were lower than those in the control group and model group (*p* < 0.05, *p* < 0.01). The levels of ALT, AST and gamma-glutamyl transferase (GGT) in serum decreased significantly(*p* < 0.05). Low density lipoprotein cholesterol (LDL-C) (71.2%–76.3%, *p* < 0.01), high-density liptein cholesterol (HDL-C) (21.6%–24.3%, *p* < 0.05), very low-density lipoprotein cholesterol (VLDL-C) (45.2%–50.0%, *p* < 0.01), triglyceride (TG) (50.6%–53.8%, *p* < 0.01), total cholesterol (TC) (45.8%, 51.0%, *p* < 0.05) significantly increased. CAT, SOD and GSH in liver increased significantly (Ye, 2018). To sum up, LJF has a significant hypoglycemic effect. LJF (4.2 g/kg) can not only reduce the level of triglyceride in serum and liver tissue of hyperlipidemic model mice, but also reduce the level of blood glucose in alloxan diabetic model mice and sucrose-induced hyperglycemia model mice (Wang et al., 2007).

Zhang et al. found that LJF polyphenol extract significantly reduced the postprandial blood glucose level of diabetic rats and inhibited the α-glucuronidase activity related to glucose absorption, with 3,5-dicaffeoyl quinic acid playing the main role, while chlorogenic acid and rutin had relatively weak inhibition (Zhang et al., 2014). Wang et al. studied the hypoglycemic and hypolipidemic effects of LJF polysaccharide on diabetic SD rats induced by streptozotocin. Compared with the last experiment, the polysaccharides had inhibitory activity on both α-amylase and α-glucosidase. The IC50 values against the inhibitory activity of the positive drug acarbose against α-amylase and α-glucosidase were 59.4 and 39.7 μg/ml, with LJF polysaccharides reaching 61.2 and 45.6 μg/ml. LJF polysaccharides (800 mg/kg) reduced serum glucose and improved insulin resistance, and increased liver and skeletal muscle glycogen content. This mechanism might be related to the recovery of pyruvate kinase and hexokinase activities. In terms of reducing blood lipid, LJF polysaccharide can reduce the content of TC, TG, LDL-C and VLDL-C and increase the content of HDL-C to achieve the effect of reducing blood lipid. The study also showed that the hypoglycemic and hypolipidemic effects of LJF polysaccharide were related to the inhibition of antioxidant enzymes ALT, AST and GGT, the increase of CAT, SOD and GSH, and the inhibition of oxidative stress response *in vivo* (Wang et al., 2017).

The above studies have shown that LJF has good therapeutic effects on diabetes and complications, coronary heart disease, atherosclerosis and other diseases caused by hyperglycemia and hyperlipidemia. Similar to the anti-tumor effect, the most studied components are still polyphenols and polysaccharides. However, most of the above studies lack positive drug comparison, and only detect the biochemical factors related to insulin resistance, glucose metabolism and lipid metabolism, and lack in-depth research on the targets, pathways and related protection mechanisms. Known antidiabetic drugs, including biguanides and sulfonylureas, are compared as positive agents. Foxo1 and Foxo3, AMPK pathway, INSR/IRS-1/PI3K/Akt/GSK-3/GLUT-4 pathway, and PI3K-Akt/PKB pathway are currently the key targets and pathways for lowering blood glucose and blood lipid. Future scholars can study the hypoglycemic and hypolipidemic effects of LJF *in vivo* and *in vitro* based on the above pathways.

### Toxic effect

There was no significant difference in acute toxicity between the aqueous extract of LD50 (72.12 g/kg) and diploid (69.92 g/kg). The administration dose is equivalent to 412 and 400 times of the safe dose of human body (body mass 60 kg). Hu Xuan et al. studied the acute toxicity of LJF and found that LJF was given four times by intra-gastric administration (Hu et al., 2015b).

Pu Hanlin et al. studied the long-term toxicity of LJF in mice. The mortality rate of LJF (3 g/100 ml) within 5 days was 70%. Before and after 5 days, the mortality rate reached 90%. The content of phosphorus, copper, magnesium and zinc in liver tissue increased significantly, the content of manganese increased slightly, the content of iron decreased significantly, the color of liver darkened and the spleen became smaller. The results show that LJF has certain toxicity to the body (Pu et al., 2011).

LJF and its active components not only have a broad-spectrum antibacterial effect, but have been used for thousands of years to treat influenza infection, as new natural food antioxidants and tumor specific drugs, but also have their own characteristics in liver protection, diabetes and other pharmacological effects. Among them, the active ingredient 3,5-dicaffeoyl quinic acid has the effect of lowering blood glucose, japoflavone D has the effect of protecting liver, 4,5-CQME and chlorogenic acid have the effect of anti-oxidation, and 3,4-dicaffeoyl quinic acid and chlorogenic acid have the effect of anti-bacteria. The components of Chinese medicine are complex, and the study of monomeric active components is an important way to obtain lead compounds and prepare drugs, as well as an effective way to promote the clinical application of Chinese medicine and in-depth study of its pharmacological mechanism. However, the current pharmacological studies on LJF are still mostly studies on alcohol extracts and water extracts. Most of the pathways identified by current studies are still incomplete and not thorough, and have only been confirmed on cells or animals alone. In addition, large doses of LJF have a certain toxicity, and there is currently a lack of research on its clinical safe dose. At present, there are few studies on antipyretic, antibacterial and antiviral effects, and almost no clear mechanism is clarified. Future scholars can continue in-depth research from the above aspects. In this study, we reviewed most of the current pharmacological research results of LJF and proposed the shortcomings and solutions, in the hope of providing a basis and reference for future research on LJF

## Conclusion and future perspectives

LJF, as a natural food and medicine with development value, has been widely used in China for thousands of years. People have developed many ways to eat them, such as raw food, tea, and steaming with other food, etc. Scholars have studied its chemical constituents and pharmacological effects deeply. So far, more than 600 components have been isolated from LJF, including essential oils, organic acids, flavonoids, iridoids, saponins, trace elements and so on. It has the functions of anti-inflammation, anti-virus, anti-bacteria, anti-oxidation, anti-tumor, protecting liver and gallbladder, anti-hyperlipidemia, anti-thrombosis, anti-allergy, immune regulation and so on. It provides great help for clinical application. However, there is still a lot of work to be done on the development and utilization of LJF:

First, compounds in plants are directly related to pharmacological effects and determine their therapeutic effects on diseases. However, the studies on the effective components of LJF are not comprehensive due to the imprecise instruments and analytical methods. The compounds isolated at present may only be a small part of LJF, and it is still recommended to continue developing new compounds in the future, which may be the key components for its efficacy. The probability of developing a drug similar to anticancer star paclitaxel is very low, but it is still hopeful. LJF has been planted on a large scale, but the annual supply still cannot keep up with the demand. And leaves and stems also exist a large number of active ingredients, and their access is easy. Therefore, more analysis of leaves and stems is also proposed in the future to expand the development of multiple sources.

Second, LJF is a medicinal and edible drug, and its therapeutic effects on various diseases have attracted attention from researchers in China and abroad. However, the molecular targets related to pharmacological effects and the pathways to exert the pharmacological basis remain to be clarified. Current research mainly focuses on preliminary animal experiments, and more *in vitro* studies at the cellular level and more comprehensive clinical applications are needed to further clarify its pharmacological mechanism of action and optimize the clinical medication standards of LJF.

Third, as a traditional Chinese medicine, LJF is widely used in medicine books, and thousands of years of experience in the clinical application of LJF has been accumulated. However, the current research on its pharmacological effects is only limited to some of the most concerned diseases, such as cancer and diabetes. This limits the application of LJF. We hope that LJF will become not only a common medicine for the above serious diseases, but also a specific medicine for other minor diseases in the future. Through in-depth exploration of the wealth recorded in ancient medical books, a broader range of modern clinical applications of LJF can be explored in the future.

Fourth, the acute, subacute and chronic toxicity evaluation of LJF is not only a prerequisite for clinical trials and the development of new drugs, but also a powerful experimental basis for its potential to become a new drug. Attention should also be paid to the toxicity study to provide a basis for the development of natural antitumor drugs and natural antioxidant cosmetics. It is believed that with the deepening of chemical and pharmacological work, more active components will be extracted and the action mechanism, action target and action channel will be clarified, in order to give full play to the clinical application value of LJF.

This study summarized the botany, ethnopharmacology, phytochemistry and pharmacological effects of LJF, hoping to comprehensively elaborate the medicinal value of LJF and promote its application. As a kind of health food with high value, LJF is worthy of further promotion and development.

## References

[B1] Bai X. Chai Y. Shi W. Li Y. Zhang T. Liu P. (2020). *Lonicera japonica* polysaccharides attenuate ovalbumin-induced allergic rhinitis by regulation of Th17 cells in BALB/c mice. J. Funct. Foods 65, 103758. 10.1016/j.jff.2019.103758

[B2] Bang B. W. Park D. Kwon K. S. Lee D. H. Jang M. Park S. K. (2019). BST-104, a water extract of *Lonicera japonica*, has a gastroprotective effect via antioxidant and anti-inflammatory activities. J. Med. Food 22 (2), 140–151. 10.1089/jmf.2018.4231 30676853

[B3] Chen J. J. Fang J. Wan J. Feng L. Zhang Y. Zhao J. H. (2009). An *in vitro* study of the anti-cytomegalovirus effect of chlorogenic acid. Her. Med. 28 (9), 1138–1141.

[B4] Chen L. Zhang H. Y. Li X. Dong J. J. Zhao T. Z. (2015). Research progress on chemical constituents of *Lonicera japonica* . Drugs Clin. 30 (1), 108–114.

[B5] Chen X. L. (2010). Effects of water extract from lonicerae japonicae flos on glycometabolism *in vitro* . Lishizhen Med. Mat. Med. Res. 21 (3), 628–629. 10.3969/j.issn.1008-0805.2010.03.056

[B6] Chinese Pharmacopoeia Commission (2020). The 2020 edition of pharmacopoeiaof the people’s Republic of China. Beijing, China: Chemical Industry Press.

[B7] Choi C. W. Jung H. A. Kang S. S. Choi J. S. (2007). Antioxidant constituents and a new triterpenoid glycoside from Flos Lonicerae. Arch. Pharm. Res. 30 (1), 1–7. 10.1007/BF02977770 17328234

[B8] Ding J. Yan G. Yang P. Zhang Y. Liu Y. (2020). Study on fingerprint and *in vitro* antiviral activity of *Lonicera japonica* polysaccharide. China Pharm. 31 (9), 1061–1067.

[B9] Duan H. Cheng M. (2009). Experimental study on pyretolysis mechanism of compatible application of Japanese Lonicerae Japonicae Flos flower bud and weeping Forsythia fruit. Mod. J. Integr. Tradit. Chin. West. Med. 18 (11), 1214–1216.

[B10] Fan Z. Li L. Bai X. Zhang H. Liu Q. Zhang H. (2019). Extraction optimization, antioxidant activity, and tyrosinase inhibitory capacity of polyphenols from *Lonicera japonica* . Food Sci. Nutr. 7 (5), 1786–1794. 10.1002/fsn3.1021 31139392 PMC6526639

[B11] Feng X. L. Xu Q. Zhao X. Y. Liu C. Chen Y. Xu W. (2013). *In vitro* antibacterial activity and *in vivo* anti-inflammatory effects of extracts from Flos Lonicerae Japonicae and its compounds. J. Shenyang Pharm. Univ. 30 (1), 35–39+62.

[B12] Gao P. (2018). The research progress on the clinical pharmacological action of Lonicera japonica. Med. Inf. 31 (23), 37–40.

[B13] Ge L. Li J. Wan H. Zhang K. Wu W. Zou X. (2018). Novel flavonoids from *Lonicera japonica* flower buds and validation of their anti-hepatoma and hepatoprotective activity *in vitro* studies. Ind. Crops Prod. 125, 114–122. 10.1016/j.indcrop.2018.08.073

[B14] Ge L. L. Xiao L. Y. Wan H. Q. Li J. M. Lv K. P. Peng S. (2019). Chemical constituents from *Lonicera japonica* flower buds and their anti-hepatoma and anti-HBV activities. Bioorg. Chem. 92, 103198. 10.1016/j.bioorg.2019.103198 31446242

[B15] Guan F. Wang H. Shan Y. Chen Y. Wang M. Wang Q. (2014). Inhibition of COX-2 and PGE2 in LPS-stimulated RAW264. 7 cells by lonimacranthoide VI, a chlorogenic acid ester saponin. Biomed. Rep. 2 (5), 760–764. 10.3892/br.2014.314 25054024 PMC4106565

[B16] Guan Z. Y. Zhao J. M. Lin Q. Z. (2009). The effect of extract of Lonicerae Japonicae Flos on bactriostasis. China Mod. doct. 47 (15), 150–151. 10.3969/j.issn.1673-9701.2009.15.075

[B17] Guang Y. L. (2018). A brief analysis of the pharmacological action and clinical application of Lonicerae Japonicae Flos. Guide China Med. 16 (35), 164–165.

[B18] Guo C. J. Shi J. Y. (2009). Study on the spectrum-effect relationship of Lonicerae Japonicae Flos's anti-influenza effect on mice. Pharmacol. Clin. Chin. Mat. Med. 25 (4), 50–52.

[B19] Han D. Chen W. Gu X. Shan R. Zou J. Liu G. (2017). Cytoprotective effect of chlorogenic acid against hydrogen peroxide-induced oxidative stress in MC3T3-E1 cells through PI3K/Akt-mediated Nrf2/HO-1 signaling pathway. Oncotarget 8 (9), 14680–14692. 10.18632/oncotarget.14747 28122344 PMC5362435

[B20] Han J. Lv Q. Y. Jin S. Y. Zhang T. T. Jin S. X. Li X. Y. (2014). Comparison of anti-bacterial activity of three types of di-O-caffeoylquinic acids in *Lonicera japonica* flowers based on microcalorimetry. Chin. J. Nat. Med. 12 (2), 108–113. 10.1016/S1875-5364(14)60017-0 24636060

[B21] Han M. H. Lee W. S. Nagappan A. Hong S. H. Jung J. H. Park C. (2016). Flavonoids isolated from flowers of *Lonicera japonica* thunb. Inhibit inflammatory responses in BV2 microglial cells by suppressing TNF‐α and IL‐β through PI3K/akt/NF‐kb signaling pathways. Phytother. Res. 30 (11), 1824–1832. 10.1002/ptr.5688 27534446

[B22] Hou Y. Jiang J. (2013). Origin and concept of medicine food homology and its application in modern functional foods. Food Funct. 4 (12), 1727–1741. 10.1039/c3fo60295h 24100549

[B23] Hu X. Li W. D. Jia L. Zhang S. F. Wu J. Qiu Z. J. (2015a). Experimental study on antimicrobial and antiviral activities of tetraploid Lonicerae Japonicae Flos *in vitro* . Mod. Chin. Med. 17 (11), 1160–1163. 10.13313/j.issn.1673-4890.2015.11.011

[B24] Hu X. Li W. D. Zhang S. F. Li O. Mo C. (2015b). Experimental study on anti-inflammatory effects and acute toxicity of aqueous extract from tetraploid Lonicerae Flos. Chin. Tradit. Herb. Drugs 46 (11), 1649–1652. 10.7501/j.issn.0253-2670.2015.11.016

[B25] Iwahashi H. Negoro Y. Ikeda A. Morishita H. Kido R. (1986). Inhibition by chlorogenic acid of haematin-catalysed retinoic acid 5, 6-epoxidation. Biochem. J. 239 (3), 641–646. 10.1042/bj2390641 3030268 PMC1147334

[B26] Kang S. Zhang J. Wei A. Wang Y. Liang H. (2014). Further textual research of Chinese materia madica-Jinyinhua. Chin. J. Pharm. Anal. 34 (11), 1922–1927. 10.16155/j.0254-1793.2014.11.002

[B27] Kao S. T. Liu C. J. Yeh C. C. (2015). Protective and immunomodulatory effect of flos Lonicerae japonicae by augmenting IL-10 expression in a murine model of acute lung inflammation. J. Ethnopharmacol. 168, 108–115. 10.1016/j.jep.2015.03.012 25819615

[B28] Kashiwada Y. Omichi Y. Kurimoto S. I. Shibata H. Miyake Y. Kirimoto T. (2013). Conjugates of a secoiridoid glucoside with a phenolic glucoside from the flower buds of *Lonicera japonica* Thunb. Phytochemistry 96, 423–429. 10.1016/j.phytochem.2013.09.021 24120297

[B29] Kim Y. Gautam S. Aseer K. R. Kim J. Chandrasekaran P. Mazucanti C. H. (2020). Hepatocyte cannabinoid 1 receptor nullification alleviates toxin-induced liver damage via NF-κB signaling. Cell Death Dis. 11 (12), 1044. 10.1038/s41419-020-03261-8 33298885 PMC7726564

[B30] Lee S. J. Shin E. J. Son K. H. Chang H. W. Kang S. S. Kim H. P. (1995). Anti-inflammatory activity of the major constituents of *Lonicera japonica* . Arch. Pharm. Res. 18 (2), 133–135. 10.1007/bf02979147

[B31] Leung H. W. C. Hour M. J. Chang W. T. Wu Y. C. Lai M. Y. Wang M. Y. (2008). P38-associated pathway involvement in apoptosis induced by photodynamic therapy with *Lonicera japonica* in human lung squamous carcinoma CH27 cells. Food Chem. Toxicol. 46 (11), 3389–3400. 10.1016/j.fct.2008.08.022 18796326

[B32] Li F. (2004). The study of Lonicera extract from water solution on ovalbumin-induced allergic mice model. J. Chongqing Med. Univ. 29 (3), 288–291. 10.3969/j.issn.1000-3606.2007.07.022

[B33] Li J. J. Ren M. L. Shang X. C. Lian X. Y. Wang H. L. (2017). Extraction of volatile oil from lonicerae japonicae flos by distillation and its component analysis. J. Henan Agric. Sci. 46 (12), 144–148. 10.15933/j.cnki.1004-3268.2017.12.027

[B34] Li K. N. Song Z. L. Jia L. L. Chu M. Xiong Z. H. (2021). Anti-inflammatory effect of Lonicerae Japonicae Flos extract on acute anterior uveitis mice induced by LPS and its mechanism. J. Jilin Univ. Med. Ed.) 47 (4), 978–983.

[B35] Li M. Y. (2010). *In vitro* anti-respiratory syncytial virus effect of the extraction of *Lonicera japonica* Thunb. J. Trop. Med. (Guangzhou) 10 (4), 420–422. 10.3969/j.issn.1672-3619.2010.04.016

[B36] Li X. P. Bai X. L. Lei L. Li X. G. Deng W. L. (2012). The antipyretic effect of lonicerae japonicae flos. Pharmacol. Clin. Chin. Mat. Med. 28 (2), 36–39.

[B37] Li X. S. (2009). Study on anti-inflammatory and protective liver effect of Lonicera japonica. Food Ind. 30 (4), 4–6.

[B38] Li Y. Chen J. Li P. (2011). Inhibitory activity of the flavonoids and phenolic acids from Jinyinhua on the xanthine oxidase. J. China Pharm. Univ. 42 (5), 407–411. 10.11665/j.issn.1000-5048.20110504

[B39] Li Y. Li W. Fu C. Song Y. Fu Q. (2020). Lonicerae japonicae flos and lonicerae flos: A systematic review of ethnopharmacology, phytochemistry and pharmacology. Phytochem. Rev. 19 (1), 1–61. 10.1007/s11101-019-09655-7 32206048 PMC7088551

[B40] Li Z. Z. (2006). Extraction method and anti-oxidation property of flavones from Lonicera Japonica Thunb's floral axises. J. Baoji Univ. Arts Ences. 2006 (02), 131–134.

[B41] Liang C. L. Liu H. Y. Cu Y. Wang L. N. Sun X. F. Zhang Y. Q. (2015). Chromatography-efficacy relation between fingerprints of lonicerae japonicae flos and its effect in scavenging DPPH free radicals. Chin. J. Exp. Tradit. Med. Formulae 21 (17), 44–46. 10.13422/j.cnki.syfjx.2015170044

[B42] Lin L. Wang P. Du Z. Wang W. Cong Q. Zheng C. (2016). Structural elucidation of a pectin from flowers of *Lonicera japonica* and its antipancreatic cancer activity. Int. J. Biol. Macromol. 88, 130–137. 10.1016/j.ijbiomac.2016.03.025 27000440

[B43] Liu B. Liu Y. H. (2013). Effects of Lonicerae lonicerae polysaccharide on splenic lymphocyte proliferation. China Prac. Med. 8 (11), 244–245. 10.14163/j.cnki.11-5547/r.2013.11.028

[B44] Liu C. Ding J. Zhou Y. Yin Z. Luo H. Kong W. (2021). Protective effects of Lonicerae Japonicae Flos against acute alcoholic liver injury in rats based on network pharmacology. China J. Chin. Mat. Med. 46 (17), 4531–4540. 10.19540/j.cnki.cjcmm.20210624.401 34581059

[B45] Liu C. P. (2009). Analysis on anti-oxidized activity of flavonoid from Honeysuckle. J. Anhui Agric. Sci. 37 (20), 9483–9484. 10.13989/j.cnki.0517-6611.2009.20.154

[B46] Liu H. Zhang D. Q. Liu S. Ma S. H. Zhang Y. L. Dong A. (2016). Study on the antioxidant activity of different extracts of the flower buds of *Lonicera japonica* . Food Res. Dev. 37 (1), 48–52.

[B47] Liu Y. F. Li L. P. Ma H. Y. Zhu L. J. Sun S. S. Hu Y. X. (2018). Research progress on the chemical constituents and pharmacological effects of Lonicera japonica thunb. J. Liaoning Univ. Nat. Sci. 45 (3), 255–262.

[B48] Liu Y. G. Liu Y. H. Jiang H. Q. (2012). Inhibitory effect and mechanism of Lonicerae japonica polysaccharide on S180 sarcoma in mice. J. Chin. Oncol. 18 (8), 584–587. 10.11735/j.issn.1671-170X.2012.8.B007

[B49] Liu Y. Wang G. L. (2011). Effect of flos lonicerae extracts on herpes simplex keratitis. Her. Med. 30 (11), 1421–1424. 10.3870/yydb.2011.11.006

[B50] Lou L. Zhou J. Liu Y. Wei Y. I. Zhao J. Deng J. (2016). Chlorogenic acid induces apoptosis to inhibit inflammatory proliferation of IL-6-induced fibroblast-like synoviocytes through modulating the activation of JAK/STAT and NF-κB signaling pathways. Exp. Ther. Med. 11 (5), 2054–2060. 10.3892/etm.2016.3136 27168850 PMC4840502

[B51] Luo H. Yang M. Tang Q. L. Hu X. Y. Willcox M. L. Liu J. P. (2021). Characteristics of registered clinical trials on traditional Chinese medicine for coronavirus disease 2019 (COVID-19): A scoping review. Eur. J. Integr. Med. 41, 101251. 10.1016/j.eujim.2020.101251 33204368 PMC7659925

[B52] Luo L. Zhang B. Ma L. Fan J. Zhu W. Guan N. (2018). Antioxidant activity *in vitro* and protective effect of flavonoids from *Lonicera japonica* Thunb. leaves on H2O2-induced toxicity in RAW264. 7 cells. Food Sci. 39 (11), 139–145. 10.7506/spkx1002-6630-201811022

[B53] Lv X. Xu W. (2022). Relationship between organic acids, luteoloside, anthocyanins and anti- oxidant activity in lonicerae japonicae flos different florescence. Mol. Plant Breed. 19 (22), 7579–7587. 10.13271/j.mpb.019.007579

[B54] Ma S. C. Liu Y. Bi P. X. Yang Y. Huang R. C. Lee H. S. (2006). Antiviral activities of flavonoids isolated from Lonicerae japonica Thunb. Chin. J. Pharm. Anal. 26 (4), 426–430.

[B55] Mi H. J. Wang Y. X. Meng J. Wang X. H. Tao Y. H. Wang Z. Z. (2015). Preliminary analysis on spectrum-efficient correlation model for anti-influenza virus of Lonicerae Japonicae Flos by partial least squares method. China J. Chin. Mat. Med. 40 (23), 4650–4654. 10.4268/cjcmm20152321 27141678

[B56] Miao Y. Xu B. Xu J. Zhang Y. Liu J. Liu Y. (2020). Establishment of HPLC fingerprint of *Lonicera japonica* and study on its anti-inflammatory spectrum-effect relationship. China Pharm. 31 (20), 2497–2502. 10.6039/j.issn.1001-0408.2020.20.12

[B57] Park H. S. Park K. I. Lee D. H. Kang S. R. Nagappan A. Kim J. A. (2012). Polyphenolic extract isolated from Korean *Lonicera japonica* thunb. Induce G2/M cell cycle arrest and apoptosis in HepG2 cells: Involvements of PI3K/akt and MAPKs. Food Chem. Toxicol. 50 (7), 2407–2416. 10.1016/j.fct.2012.04.034 22561682

[B58] Patil M. P. Bayaraa E. Subedi P. Piad L. L. A. Tarte N. H. Kim G. D. (2019). Biogenic synthesis, characterization of gold nanoparticles using *Lonicera japonica* and their anticancer activity on HeLa cells. J. Drug Deliv. Sci. Technol. 51, 83–90. 10.1016/j.jddst.2019.02.021

[B59] Peng L. Mei S. Jiang B. Zhou H. Sun H. (2000). Constituents from *Lonicera japonica* . Fitoterapia 71 (6), 713–715. 10.1016/s0367-326x(00)00212-4 11077184

[B60] Peng S. Huo X. Q. Huo M. Q. Liu Y. N. Zhang Y. L. Qiao Y. J. (2020). Study on efficacy markers of heat-clearing and detoxifying effect of Lonicerae Japonicae Flos based on systematic traditional Chinese medicine. China J. Chin. Mat. Med. 45 (14), 3275–3281. 10.19540/j.cnki.cjcmm.20200210.403 32726040

[B61] Pi J. H. Tan J. Hu Z. T. Xiang D. B. (2015). Effects of Lonicera Japonica flavone on immunomodulation in mice. Chin. J. Appl. Physiol. 31 (1), 89–92. 10.13459/j.cnki.cjap.2015.01.026 26016250

[B62] Pu H. L. Jiang H. Chen R. R. (2011). Toxicity of lonicerae japonicae flos and dry ginger on mice. Pract. Pharm. Clin. Rem. 14 (4), 277–278. 10.14053/j.cnki.ppcr.2011.04.007

[B63] Qi L. Chen C. Li P. (2009). Structural characterization and identification of iridoid glycosides, saponins, phenolic acids and flavonoids in Flos Lonicerae Japonicae by a fast liquid chromatography method with diode‐array detection and time‐of‐flight mass spectrometry. Rapid Commun. Mass Spectrom. 23 (19), 3227–3242. 10.1002/rcm.4245 19725056

[B64] Rajivgandhi G. N. Chackaravarthi G. Ramachandran G. Manoharan N. Ragunathan R. Siddiqi M. Z. (2022). Synthesis of silver nanoparticle (Ag NPs) using phytochemical rich medicinal plant *Lonicera japonica* for improve the cytotoxicity effect in cancer cells. J. King Saud Univ. - Sci. 34 (2), 101798. 10.1016/j.jksus.2021.101798

[B65] Ren M. T. Chen J. Yue S. Sheng L. S. Ping L. Qi L. W. (2008). Identification and quantification of 32 bioactive compounds in Lonicera species by high performance liquid chromatography coupled with time-of-flight mass spectrometry. J. Pharm. Biomed. Anal. 48 (5), 1351–1360. 10.1016/j.jpba.2008.09.037 18977626

[B66] Shang X. Pan H. Li M. Miao X. Ding H. (2011). *Lonicera japonica* thunb.: Ethnopharmacology, phytochemistry and pharmacology of an important traditional Chinese medicine. J. Ethnopharmacol. 138 (1), 1–21. 10.1016/j.jep.2011.08.016 21864666 PMC7127058

[B67] Shi Z. Liu Z. Liu C. Wu M. Su H. Ma X. (2016). Spectrum-effect relationships between chemical fingerprints and antibacterial effects of Lonicerae Japonicae Flos and Lonicerae Flos base on UPLC and microcalorimetry. Front. Pharmacol. 7, 12. 10.3389/fphar.2016.00012 26869929 PMC4735347

[B68] Son K. H. Park J. O. Chung K. C. Chang H. W. Kim H. P. Kim J. S. (1992). Flavonoids from the aerial parts of *Lonicera japonica* . Arch. Pharm. Res. 15 (4), 365–370. 10.1007/bf02974114

[B69] Song J. H. (2011). Study on antipyretic and anti-inflammatory effect of lonicera japonica. Chongqing Med. 40 (25), 2552–2553. 10.3969/j.issn.1671-8348.2011.25.024

[B70] Song Y. L. Ni F. Y. Zhao Y. W. Xie X. Wang W. Z. Wang Z. Z. (2014). Research progress on chemical constituents from Lonicerae Flos. Chin. Tradit. Herb. Drugs 45 (24), 3656–3664. 10.7501/j.issn.0253-2670.2014.24.027

[B71] Song Y. L. Wang H. M. Ni F. Y. Wang X. J. Zhao Y. W. Huang W. Z. (2015). Study on anti-inflammatory activities of phenolic acids from Lonicerae Japonicae Flos. Chin. Tradit. Herb. Drugs 46 (4), 490–495. 10.7501/j.issn.0253-2670.2015.04.006

[B72] Sun C. Teng Y. Li G. Yoshioka S. Yokota J. Miyamura M. (2010). Metabonomics study of the protective effects of *Lonicera japonica* extract on acute liver injury in dimethylnitrosamine treated rats. J. Pharm. Biomed. Anal. 53 (1), 98–102. 10.1016/j.jpba.2010.03.015 20371156

[B73] Tan Z. W. Xia W. Yu Y. L. Center S. (2018). Research progress on chemical constituents and pharmacology of honeysuckle. J. Anhui Agric. Sci. 46 (9), 26–28+123. 10.13989/j.cnki.0517-6611.2018.09.008

[B74] Teng Y. Luo S. X. Guo Y. X. Tan T. Zhang Y. Sun C. H. (2016). Protective effects of Lonicera japonica extract on acute liver injury in rats by the method of metabolomic. Food Res. Dev. 37 (4), 29–34.

[B75] Teng Y. Tan T. Luo S. X. Zhang Y. Xin H. U. Jiang D. T. (2014). Protective effects of Lonicera japonica extract on acute liver injury in rats and antioxidant activity. Food Res. Dev. 35 (4), 57–59. 10.3969/j.issn.1005-6521.2014.24.015

[B76] Tian L. Jiang B. P. Miao H. E. Shen L. I. Fan X. F. (2014). Study on antioxidant activity of water extracts from different proportions of Lonicerae Japonicae Flos flower and wild Lonicerae Japonicae Flos flower *in vitro* . Pract. Pharm. Clin. Rem. 17 (10), 1290–1294. 10.14053/j.cnki.ppcr.2014.10.018

[B77] Tu S. P. Huang D. Huang H. Wu D. Wu C. L. Dai J. (2020). The *in vivo* effects of *Lonicera japonica* Thunb on CYP3A enzyme activity and gene expression in rats. J. Chengdu Med. Coll. 15 (2), 204–207+231. 10.3969/j.issn.1674-2257.2020.02.017

[B78] Tzeng T. F. Tzeng Y. C. Cheng Y. J. Liou S. S. Liu I. M. (2015). The ethanol extract from *Lonicera japonica* Thunb. regresses nonalcoholic steatohepatitis in a methionine-and choline-deficient diet-fed animal model. Nutrients 7 (10), 8670–8684. 10.3390/nu7105423 26506376 PMC4632443

[B79] Wang B. L. Gao Y. Zhao X. X. (2015). Study on Lonicera japonica thunb antiviral effect *in vitro* . Liaoning J. Tradit. Chin. Med. 42 (8), 1495–1497. 10.13192/j.issn.1000-1719.2015.08.047

[B80] Wang D. Zhao X. Liu Y. (2017). Hypoglycemic and hypolipidemic effects of a polysaccharide from flower buds of *Lonicera japonica* in streptozotocin-induced diabetic rats. Int. J. Biol. Macromol. 102, 396–404. 10.1016/j.ijbiomac.2017.04.056 28419828

[B81] Wang G. Zhu X. Wang J. Jia W. Yuan Y. Nan P. (1992). Analysis of chemical constituent of essential oil in Lonicera japonnica Thunb. cultivated on the northern plain of Henan Province. China J. Chin. Mat. Med. 17 (5), 268–270, 319. 1418559

[B82] Wang L. Jiang Q. Hu J. Zhang Y. Li J. (2016a). Research progress on chemical constituents of Lonicerae japonicae flos. Biomed. Res. Int. 2016, 8968940. 10.1155/2016/8968940 27403439 PMC4923575

[B83] Wang Q. Chen D. H. Deng W. L. (2007). Effect of extract of Lonicerae Japonicae Flos on blood lipid and blood glucose. Pharmacol. Clin. Chin. Mat. Med. 23 (3), 40–42. 10.3969/j.issn.1001-859X.2007.03.021

[B84] Wang Q. Zhu X. X. Zhang C. B. Ni W. P. Wu X. T. (2008a). Experiment study on lonicerae japonicae flos extract against bacteria. Chin. J. Med. Guide 10 (9), 1428–1430.

[B85] Wang T. Yang B. Guan Q. Chen X. Zhong Z. Huang W. (2019). Transcriptional regulation of *Lonicera japonica* Thunb. during flower development as revealed by comprehensive analysis of transcription factors. BMC Plant Biol. 19 (1), 198. 10.1186/s12870-019-1803-1 31088368 PMC6518806

[B86] Wang X. (2015). Analysis of medicinal components and pharmacological action of *Lonicera japonica* Thunb. Asia-pac. Tradit. Med. 11 (18), 30–31. 10.11954/ytctyy.201518015

[B87] Wang Y. Chen W. Zhong S. Zhang H. Xue M. Gu B. (2016b). Effect of heat-clearing and detoxifying health function of lonicera japonica in rats based on metabonomics. J. Chin. Med. Mat. 39 (5), 1129–1133. 10.13863/j.issn1001-4454.2016.05.045 30133198

[B88] Wang Y. Xue X. Zhang F. Qing X. Liang X. (2008b). Separation and analysis of volatile oil from *Lonicera japonica* thunb. World Sci. Technol. 10 (6), 45–55. 10.1016/s1876-3553(10)60004-x

[B89] Wang Z. Xiao C. Y. Tian L. Zheng Q. F. (2010). [Discussion on herbal textual research of Flos Lonicerae]. J. Chin. Mat. Med. 35 (8), 1086–1088. 10.4268/cjcmm20100831 20617698

[B90] Wu J. Wang C. Yu H. (2019). Chemical constituents and pharmacological effect of lonicerae japonicae flos. Chin. J. Exp. Tradit. Med. Formulae 25 (4), 225–234. 10.13422/j.cnki.syfjx.20190408

[B92] Xia W. Yu Y. Yang H. Tan Z. Xu L. Dong W. (2017). Research advances on chemical constituent and pharmacology effects of lonicerae japonicae flos. J. Anhui Agric. Sci. 45 (33), 126–127+165. 10.13989/j.cnki.0517-6611.2017.33.042

[B93] Xia Y. Li D. Pei Z. Zhang Y. (2012). Review on the chemical constituents of the flower buds of *Lonicera japonica* . Mod. Chin. Med. 14 (4), 26–32. 10.13313/j.issn.1673-4890.2012.04.016

[B94] Xiao L. Liang S. Ge L. Wan H. Wu W. Fei J. (2020). 4, 5-di-O-caffeoylquinic acid methyl ester isolated from *Lonicera japonica* Thunb. targets the Keap1/Nrf2 pathway to attenuate H2O2-induced liver oxidative damage in HepG2 cells. Phytomedicine 70, 153219. 10.1016/j.phymed.2020.153219 32361557

[B95] Xiao M. Tan H. J. Li X. H. Kang H. P. (2013). Study on essential oil from leaf of Lonicera japonic by GC-MS. J. Anhui Agric. Sci. 41 (03), 947+996. 10.13989/j.cnki.0517-6611.2013.03.105

[B96] Xu M. Y. Hu J. J. Shen J. Wang M. L. Zhang Q. Q. Qu Y. (2014). Stat3 signaling activation crosslinking of TGF-β1 in hepatic stellate cell exacerbates liver injury and fibrosis. Biochim. Biophys. Acta 1842 (11), 2237–2245. 10.1016/j.bbadis.2014.07.025 25092172

[B97] Xu X. B. Xu P. Mao X. F. Li J. F. Ren M. (2018). Content and antioxidant activity of three active components in different organs of lonicerae japonicae thunb. Sci. Technol. Food Ind. 39 (13), 41–45. 10.13386/j.issn1002-0306.2018.13.008

[B98] Xu X. Y. Miao F. Wang X. D. Gao Y. F. Zhang Z. Y. Su S. Z. (2020). The effect and mechanism of *Lonicera japonica* total flavones on hepatic fibrosis induced by carbon tetrachloride in rats. J. Taishan Med. Coll. 41 (1), 1–4. 10.3969/j.issn.1004-7115.2020.01.001

[B99] Yan S. H. Miao H. Huang Z. L. Ji L. L. (2021). Protective effects of extract combination of Lonicerae Japonicae Flos and Forsythiae Fructus against CCl 4 -induced liver fibrosis in mice. Acad. J. Shanghai Univ. Tradit. Chin. Med. 35 (2), 50–56. 10.16306/j.1008-861x.2021.02.010

[B100] Yan X. L. Meng A. P. Pu S. B. (2016). Research progress on activities of anti-inflammation and immunity from the flower of *Lonicera japonica* thunb. Chin. Wild Plant Resour. 35 (2), 41–44. 10.3969/j.issn.1006-9690.2016.02.012

[B101] Yang J. Li Y. C. Zhou X. R. Xu X. J. Fu Q. Y. Liu C. Z. (2018). Two thymol derivatives from the flower buds of *Lonicera japonica* and their antibacterial activity. Nat. Prod. Res. 32 (18), 2238–2243. 10.1080/14786419.2017.1371153 28868923

[B102] Yang Q. R. Zhao Y. Y. Hao J. B. Li W. D. (2016). Research progress on chemical constituents and their differences between lonicerae japonicae flos and lonicerae flos. China J. Chin. Mat. Med. 41 (7), 1204–1211. 10.4268/cjcmm20160708 28879732

[B103] Yang R. Fang L. Li J. Zhao Z. Zhang H. Zhang Y. (2019). Separation of five iridoid glycosides from Lonicerae japonicae flos using high-speed counter-current chromatography and their anti-inflammatory and antibacterial activities. Molecules 24 (1), 197. 10.3390/molecules24010197 PMC6337566 30621066

[B104] Yang S. K. (2019). Study on chemical constituents and biological activities of Lonicerae Japonicae Flos. Chem. Eng. Des. Commun. 45 (11), 149+177. 10.3969/j.issn.1003-6490.2019.11.098

[B105] Yao C. L. Wei W. L. Zhang J. Q. Bi Q. R. Li J. Y. Khan I. (2022). Traditional Chinese medicines against COVID-19: A global overview. World J. Tradit. Chin. Med. 8 (3), 279. 10.4103/2311-8571.353502

[B106] Ye Q. H. (2018). Experimental studies on hypoglycemic effects of extracts from Lonicerae Japonicae Flos. Clin. J. Chin. Med. 10 (19), 4–7. 10.3969/j.issn.1674-7860.2018.19.002

[B107] Yin H. M. Lv X. Y. Xiao W. (2010). Study on preparation process optimization and immune activity of Lonicerae lonicerae polysaccharide. China J. Chin. Mat. Med. 35 (4), 453–455.

[B108] Zeng H. Q. Mao L. Jin Y. H. Li S. T. Xu A. (2022). *In vitro* study of the effects of Lonicerae Japonicae Flos on Streptococcus mutans UA159. J. Prev. Treat. Stomatol. Dis. 30 (8), 542–548. 10.12016/j.issn.2096-1456.2022.08.002

[B109] Zhang M. L. Li F. Liu W. Shi L. Yang J. X. (2016). Research progress on the anti-virus effect of Lonicerae Flos. J. Liaoning Univ.Traditi. Chin. Med. 18 (9), 156–158. 10.13194/j.issn.1673-842x.2016.09.048

[B110] Zhang W. Huang L. Q. Li C. X. Li J. Zhang R. X. (2014). Literature study on species of honeysuckle flower. China J. Chin. Mat. Med. 39 (12), 2239–2245. 10.4268/cjcmm20141218 25244752

[B111] Zhang W. W. Ren Y. Zhang R. C. Hao G. Z. (2020). Study on the effects of volatile oil from Flos Lonicerae and leaves of *Lonicera japonica* on lymphocyte proliferation in mice. J. Pharm. Res. 39 (4), 198–201+224. 10.13506/j.cnki.jpr.2020.04.003

[B112] Zhang Z. Shen H. Sun Y. Ding W. (2019). Studied on phenolic acids extracting of *Lonicera japonica* Thunb. and its antimicrobial Effect. Chin. J. Ethnomed. Ethnopharm 28 (16), 27–29.

[B113] Zhao A. Y. (2018). A brief analysis of the pharmacological action and clinical application of Lonicerae Japonicae Flos. Contemp. Med. Symp. 16 (4), 53–54.

[B114] Zhao M. M. Zhao Z. G. Liu Q. Liu W. Wang X. Zhao H. Q. (2021). Rapid discovery of trace antioxidants in lonicerae japonicae flos by 2D-HPLC-DPPH-ESI-TOF/MS. Chin. Tradit. Herb. Drugs 52 (11), 3193–3200. 10.7501/j.issn.0253-2670.2021.11.005

[B117] Zhou X. Dong Q. Kan X. Peng L. Xu X. Fang Y. (2018). Immunomodulatory activity of a novel polysaccharide from *Lonicera japonica* in immunosuppressed mice induced by cyclophosphamide. PloS one 13 (10), e0204152. 30296293 10.1371/journal.pone.0204152 PMC6175272

[B115] Zhou X. P. Li Z. M. Liu Z. J. Yang D. Q. Qiu H. Lin M. (2011). Effect of flos lonicerae on immunologic function of rats. Pract. Prev. Med. 18 (2), 214–216. 10.3969/j.issn.1006-3110.2011.02.008

[B116] Zhuang L. Zhang C. Ali M. S. (2013). Advances in pharmacological action and clinical application of flos lonicerae. Liaoning J. Tradit. Chin. Med. 40 (2), 378–380. 10.13192/j.ljtcm.2013.02.192.zhuangl.067

